# Deletion of *Socs3* in LysM^+^ cells and *Cx3cr1* resulted in age-dependent development of retinal microgliopathy

**DOI:** 10.1186/s13024-021-00432-9

**Published:** 2021-02-18

**Authors:** Xuan Du, Rosana Penalva, Karis Little, Adrien Kissenpfennig, Mei Chen, Heping Xu

**Affiliations:** The Wellcome-Wolfson Institute for Experimental Medicine, School of Medicine, Dentistry & Biomedical Sciences, Queen’s University Belfast, 97 Lisburn Road, BT9 7BL Belfast, Northern Ireland, UK

**Keywords:** Aging, Primary microglial dysfunction, Retinal degeneration, Microgliopathy, Neuroinflammation, Neuron-microglial interaction, Cytokines, Chemokines

## Abstract

**Background:**

We generated a mouse model of primary microglial dysfunction by deleting two negative immune regulatory genes, *Cx3cr1* and *Socs3* (in LysM^+^ cells). This study aimed to understand how primary microglial dysfunction impacts retinal neurons during aging.

**Methods:**

The *LysMCre-Socs3*^*fl/fl*^*Cx3cr1*^*gfp/gfp*^ double knockout (DKO), *LysMCre-Socs3*^*fl/fl*^, *Cx3cr1*^*gfp/gfp*^ and *Socs3*^*fl/fl*^ mice were maintained up to 12 months. Eyes were collected and processed for immunohistochemistry of IBA-1, cone arrestin, secretagogin, PKCα and GABA. Brain microglia from DKO and WT mice were stimulated with LPS + IFN-γ or IL-4. The expression of TNF-α, IL-1β, IL-6, iNOS, IL-12p40, IL-23p19, CCL2, CCL5, CXCL2, IL-10, CD206 and Arg1 were examined by qRT-PCR and protein production was measured by Luminex assay. Retinal explants from C57BL/6 J mice were co-cultured with microglia from DKO or WT mice for 24 h, after which the number of cone arrestin^+^ cells in retinal flatmount were quantified.

**Results:**

In 3–5 month old mice, the number of microglia in retinal ganglion cell layer (GCL) and inner plexiform layer (IPL) were comparable in all strains of mice. The DKO mice had a significantly higher number of microglia in the outer plexiform layer (OPL) but significantly lower numbers of cone arrestin^+^, secretagogin^+^ and GABA^+^ cells compared to *Socs3*^*fl/fl*^ and single KO mice. During aging, 57% of the DKO mice died before 12 months old. The 10–12 months old DKO mice had significantly higher numbers of microglia in GCL/IPL and OPL than age-matched *Socs3*^*fl/fl*^ and single KO mice. The aged DKO mice developed retinal pigment epithelial (RPE) dysmorphology accompanied by subretinal microglial accumulation. The number of photoreceptors, bipolar cells (Secretagogin^+^ or PKCα^+^) and GABA^+^ amacrine cells was significantly lower in aged DKO mice compared to age-matched *Socs3*^*fl/fl*^ and single KO mice. Microglia from DKO mice showed significantly higher levels of phagocytic activity and produced higher levels of TNF-α, IL-6, CCL2, CCL5, CXCL2 and CXCL10 compared to microglia from *Socs3*^*fl/fl*^ mice. Co-culture of retinal explants with LPS + IFN-γ or IL-4 pre-treated DKO microglia significantly reduced cone photoreceptor survival.

**Conclusions:**

The *LysMCre*-*Socs3*^*fl/fl*^*Cx3cr1*^*gfp/gfp*^ DKO mice displayed primary microglial dysfunction and developed age-related retinal microgliopathy characterized by aggragated microglial activation and multiple retinal neuronal and RPE degeneration.

**Trial registration:**

Not applicable. The article does not contain any results from human participants.

**Supplementary Information:**

The online version contains supplementary material available at 10.1186/s13024-021-00432-9.

## Background

Microglia, the resident innate immune cells of the central nervous system (CNS), which includes the retina, play an important role in safeguarding neurons from exogenous and endogenous insults [1–3]. Microglia can detect damage-associated molecular patterns (DAMPs) released by diseased neurons or pathogen-associated molecular patterns (PAMPs) from invading microbes with pattern recognition receptors (PRRs) such as toll-like receptors (TLRs), NOD-like receptors (NLRs) and RIG-like receptors (RLRs). PRR activation can lead to a cascade of signalling transduction and upregulation of a variety of inflammatory gene expression. The primary role of microglial activation is to eliminate the insults (e.g., dead cells or invading pathogens), which therefore is neuroprotective [4, 5]. Under certain conditions, active microglia can promote neuronal regeneration [6]. Uncontrolled microglial activation, however, is detrimental and contributes to various neurodegenerative diseases such as Alzheimer’s disease [7, 8], Parkinson’s disease [9], age-related macular degeneration (AMD) [10–12], diabetic retinopathy [13, 14], inherited retinal degeneration [15] and glaucoma [16]. Targeting microglial activation is considered to be an effective approach for the management of neurodegenerative diseases [17–19]. Until now, the attempts to control microglial activation with general immune suppression (e.g., with steroids or immune suppressive drugs) have achieved limited clinical success [20–22]. In most conditions, microglial activation is secondary to other pathological processes that damage the CNS or retina. Thus, activated microglia may both exacerbate pathology and aid tissue repair. To develop effective microglia-targeted therapy, it is important that we understand the molecular pathways specific to the neurotoxic effects of activated microglia.

Microglial activation is tightly controlled at multiple levels. Microglia express various receptors (e.g., CX3CR1, SIRPα and CD200R) that can sense inhibitory molecules from surrounding neurons (e.g., CX3CL1, CD47 and CD200 from ganglion cells and amacrine cells of the retina) [23–25]. This neuron-to-microglia communication prevents overt microglial activation and protects neurons [23, 24]. For example, when the CX3CL1-CX3CR1 pathway is disrupted, microglial activation and retinal degeneration is increased in diabetes [26] or acute stress conditions [27]. Microglial activation is also regulated at intracellular levels. When the PRRs or cytokine receptors of microglia are activated, the intracellular signalling transduction pathways are regulated by various immune checkpoint proteins. The Janus kinase (JAK) and signal transducer and activator of transcription protein (STAT) signalling pathway of the cytokine receptors are negatively regulated by the suppressor of cytokine signaling (SOCS) proteins [28, 29]. Studies have shown that deletion of *Socs3* in LysM^+^ cells (*LysMCre*-*Socs3*^*fl/fl*^) did not affect microglia in normal conditions, yet enhanced the inflammatory response in the CNS in experimental autoimmune encephalomyelitis and increased demyelination [30]. The inflammatory response in experimental autoimmune uveoretinitis was also exaggerated in *LysMCre*-*Socs3*^*fl/fl*^ mice accompanied by severe retinal degeneration and angiogenesis [31]. The *LysMCre*-*Socs3*^*fl/fl*^ mice also developed more severe diabetic retinopathy compared with WT mice [32]. These results suggest that SOCS3 and CX3CR1 are both critically involved in regulating microglial activation in disease conditions.

During aging, oxidative stress accumulates in the retina, particularly the macula. As a result, a low-grade parainflammation presented as mild microglial activation, subretinal migration and complement activation is initiated [33]. This parainflammatory response, when controlled properly, is beneficial and the retina may age healthily. When the parainflammatory response is dysregulated, it may turn into chronic inflammation leading to retinal degeneration [11]. We hypothesized that the combined deletion of two negative immune regulators in microglia, CX3CR1 in the cell surface and SOCS3 in the cytosol, may subject the cells to uncontrolled activation in response to age-mediated low-levels of oxidative insults. In this study, we generated a *Cx3cr1* and *Socs3* double knockout (*LysMCre*-*Socs3*^*fl/fl*^*Cx3cr1*^*gfp/gfp*^) mouse line (referred to as DKO mice in this paper), in which *Cx3cr1* was deleted globally and *Socs3* was deleted in LysM^*+*^ cells using the Cre/LoxP technology (Additional file 1B). In the retina, these two genes were depleted only in innate immune cells, predominately microglia. We found that the DKO mice presented with significant microglial dysfunction, and with ageing, microglia were activated accompanied by substantial neuronal degeneration – a typical example of primary microglial disorder-mediated pathology i.e., “microgliopathy” [34].

## Materials and methods

### Animals and study design

C57BL/6 J wild type, *Socs3*^*fl/fl*^ [31], *LysMCre*-*Socs3*^*fl/fl*^ [31], *Cx3cr1*^*gfp/gfp*^ [27, 35] and *LysMCre-Socs3*^*fl/fl*^*Cx3cr1*^*gfp/gfp*^ DKO mice (all in C57BL/6 J background) were used in the study. The *Socs3*^*fl/fl*^ mice served as WT control. The *LysMCre*-*Socs3*^*fl/fl*^ mice were obtained by crossbreeding *Socs3*^*fl/fl*^ mice with *LysMCre* mice [31]. The *Cx3cr1*^*gfp/gfp*^ mice were provided by Steffen Jung (Weizmann Institute of Science, Rehovot, Israel). The *LysMCre*-*Socs3*^*fl/fl*^ mice and *Cx3cr1*^gfp/gfp^ mice were used to generate heterozygous *LysMCre*^*+/−*^*Socs3*^*fl/−*^*Cx3cr1*^*+/gfp*^ mice. Subsequent crossings between offspring mice allowed the generation of *LysMCre-Socs3*^*fl/fl*^*Cx3cr1*^*gfp/gfp*^ DKO (Abbreviated as DKO in the following context) mice (Additional file 1). The genotype of the DKO mice was confirmed by PCR (Additional file 1C). Mouse strains used for this study were listed in additional file 2. All animal procedures were conducted in accordance with the Animals (Scientific Procedures) Act of the UK Home Office (1986) and were approved by the Animal Welfare and Ethics Review Body of Queen’s University Belfast. All mice were housed at the Biological Research Unit at Queen’s University Belfast in a 12-h light / dark cycle with free access to water and food.

### In vivo retinal investigations

#### Fundus investigation

Fundus images were obtained. Animals were anesthetized with an intraperitoneal injection of 60 mg/kg ketamine hydrochloride (Fort George Animal Centre, Southampton, UK) and 5 mg/kg xylazine (Pharmacia & Veterinary Products, Kiel, Germany). The pupils were dilated with a drop of tropicamide and phenylephrine (Chauvin, Essex, UK). Images were captured with a Nikon D90 camera via an endoscope [36, 37] or the Micron IV system (Phoenix Research Labs, Pleasanton, CA, USA). Retinal green fluorescent images in *Cx3cr1*^*gfp/gfp*^ and DKO mice were acquired using the Micron IV system. The illumination settings and the gain were consistent in each subject. Images were saved in TIFF format.

#### Electroretinopathy (ERG)

Ganzfeld ERG was performed using a Diagosys Espion System (Diagnosys Technologies, MA, USA) using the protocol described in our previous publication [38]. In brief, mice were dark-adapted overnight and anaesthetised with 60 mg/kg ketamine hydrochloride and 5 mg/kg xylazine and the pupils dilated and moisturised as described above. For each animal, 8 light intensities (from 0.008 to 25 cd.s/m^2^) were applied. A-wave, b-wave amplitudes and oscillatory potential (OPs, summed amplitude of wavelets 2–5) were measured using the Espion analysis software.

### Retinal and RPE/Choroidal flatmount preparation and staining

The eyes were collected and fixed with 2% paraformaldehyde (PFA, Agar Scientific Ltd. Cambridge, UK) for 2 h at room temperature. The anterior segment (cornea, iris and ciliary body, and lens) of the eye was removed. Retinal tissue was carefully removed from the eyecup. Retinal tissues and RPE/choroid/sclera eye-cups were processed for flatmount staining using the protocol described in our previous paper [36, 37]. The antibodies used in the study were detailed in Table 1. All samples were examined by Dmi8 fluorescence microscopy (Leica Microsystems CMS, Mannheim, Germany). Images were analysed using ImageJ (National Institutes of Health, Bethesda, MD, USA).

**Table 1:** Antibodies and binding protein/peptide

	Name	Dilution	Company	Host/Type
Antigen	IBA-1	1:200	Wako	Rabbit
	Cone arrestin	1:1000	Chemicon	Rabbit
	PKCα	1:500	Santa Cruz	Rabbit
	Secretagogin	1:500	Biovendor R&D	Sheep
	GFAP	1:250	Abcam	Rabbit
Protein/peptide	Isolectin B4	1:200	Vector	
	Alexa Fluor™ 594 Phalloidin	1:100	ThermoFisher	
Secondary Ab	Alexa Fluor 594	1:400	Invitrogen	Donkey anti-rabbit
	Alexa Fluor 488	1:400	Invitrogen	Donkey anti-rabbit
	Streptavidin, Alexa Fluor™ 594	1:400	Invitrogen	

### H&E staining and immunohistochemistry

Eyes were fixed as described above and processed for 6 μm-thick paraffin sections. Eye sections were dewaxed using xylene, dehydrated by ethanol and rehydrated by H_2_O. Sections were then processed for either H&E staining or immunohistochemistry. Antigen retrieval was carried out using 0.05% citraconic anhydride buffer (Sigma-Aldrich, St. Louise, Missouri, USA) in a 95 °C water bath for 30 mins. Sections were permeabilized with 0.5% Triton 100-X in PBS for 15 min at room temperature and blocked using 5% BSA for 1 h. Sections were then incubated with primary antibodies (Table 1) overnight at 4 °C. After thorough washes, samples were incubated with secondary antibody for 1 h at room temperature. Samples were mounted with Vectashield mounting medium containing DAPI (Vector Laboratories) and examined by fluorescence microscopy (Leica Dmi8).

### Quantification of microglial number in retinal and RPE/choroid flatmounts

Z-stack images (20X) with 1 μm intervals were taken from the mid-central part of the retina in each flatmount (4 images/flatmount). Cells in three different layers, i.e., GCL + IPL, OPL, outer nuclear layer + photoreceptor layer (ONL + PRL) of each z-stack image were quantified by Fiji software (National Institutes of Health, Bethesda, MD). The numbers were normalized to per mm^2^ retinal area. For RPE/choroid flatmounts, images were obtained for the entire tissue and the total number of microglia was manually counted.

### Quantification of retinal neurons

Fluorescent images were obtained to quantify the number of individual retinal neurons and neuronal structure. Three sections around the optic disc were used for retinal neuronal investigations, with 3–5 eyes used in each group. Four images were taken from the mid-central part of the retina in each eye section. The settings of fluorescence microscope remained constant between images and images were analyzed using FIJI software (National Institutes of Health, Bethesda, MD). The following retinal cells were quantified and analysed using the methods established in our groups [38, 39]: 1) Rod and cone photoreceptor (cone arrestin^+^) cell count; 2) Cone photoreceptor segment length; 3) Cone-bipolar (PKCα^+^) cell number; 4) Rod-bipolar (secretagogin^+^) cell number; and 5) GABA^+^ amacrine cell number. Cell numbers were averaged and normalised. The quantification was confirmed by two independent researchers.

### Quantification of microglial cells and RPE morphology in aged DKO RPE/choroid flatmounts

Five fluorescent images (40X objective lens) were taken from each RPE/choroidal flatmount. The number of microglial cells and the morphology of RPE in aged DKO mice were quantified. 6 eyes per group were used in this study. FIJI ImageJ plug-in Bio-voxxel software [40] was used to outline the shape of each RPE cell. For each RPE cell, the area and perimeter were measured using FIJI and the shape factor was calculated by the eq. 4*π*∗*Area/Perimeter*^2^ [41]. The shape factor value ranges from 0 to 1, with one indicating a perfect circular cell, and zero indicating an elongated cell. The shape factor of a perfect hexagonal cell (eg. a RPE cell) is around 0.9 [42]. The relationship between microglial cell number and the shape factor of the RPE was analyzed by linear regression using SPSS statistics software (IBM, Chicago, IL, USA). The quantification was confirmed by two independent researchers.

### Primary microglial cell culture and treatments

Microglia were cultured from 4 to 6 week old *Socs3*^*fl/fl*^ and DKO mice using a previously described protocol [43]. Briefly, smashed brain tissue suspensions were filtered through a 70 μm cell strainer (BD Falcon, BD Biosciences). Then the cell suspensions were centrifuged at 600 g for 8 min. The cell pellet was re-suspended in media containing 10% fetal calf serum (FCS), 20 ng/ml M-CSF (Bio-techne, Minneapolis, Minnesota, US), 1% penicillin/streptomycin, 1 mM glutamine in DMEM/F12 (all from Thermo Fisher Scientific, Waltham, MA, USA), and seeded in a 6-well plate (Thermo Fisher Scientific). Floating cells were removed 3 days later and media were changed every 3 days until cells reached 90% confluence. The phenotype of the cells was confirmed by their CD11b and IBA-1 expression (> 90%). The microglia were then treated with 1) M1, or pro-inflammatory stimuli by adding 100 ng/ml IFN-γ (Bio-Techne) and 50 ng/ml LPS (Sigma-Aldrich) or 2) M2, or anti-inflammatory stimuli by adding 20 ng/ml IL-4 (Bio-Techne) overnight. The cells were collected for real-time RT-PCR for cytokine/chemokine gene expression and the supernatants were collected for Luminex assay.

### Phagocytosis assay

Microglia from *Socs3*^*fl/fl*^ or DKO mice were seeded into a black-walled 96-well plate at the density of 1 × 10^5^/well. The phagocytosis assay was performed using 1) the pHrodo™ Red *E. coli* BioParticles™ and 2) *Escherichia coli* (K-12 strain) Alexa Fluor 594 BioParticles™ (both from Thermo Fisher Scientific) following the manufacturer’s instructions. Culture medium with Bioparticles alone was served as background control. The pHrodo Red *E.coli* BioParticles do not show any fluorescence at neutral pH, but are fluorescence brightly in acidic environments (e.g., engulfed into lysosome after phagocytosis). The fluorescence intensity was measured at 0.5 h, 1 h, 2 h and 3 h after pHrodo Red *E.coli* BioParticles incubation using Fluostar Omega microplate reader (BMG Labtech, Ortenberg, Germany) with 550 nm excitation wavelength and a 600 nm emission filter. Alexa Fluor 594 conjugated *E.coli* was washed with PBS after 0.5 h incubation, and fluorescence intensity was measured with the same wavelength. Phagocytosis was calculated by subtracting the average fluorescence intensity of the no-cell background-control wells from experimental wells. Representative images were taken using the Leica DMi8 microscope.

### Reverse transcription and real-time RT-PCR

Total RNA was extracted from retinal tissues by RNeasy Mini kit (Qiagen Ltd., Crawley, UK) according to the manufacturer’s instructions. The quantity and quality of RNA were determined using a NanoDrop ND-1000 spectrophotometer (NanoDrop Technologies, Wilmington, DE). The same amount of RNA was applied for reverse transcription using SuperScrip™ II Reverse Transcriptase kit and random primers (Invitrogen). Real-time RT-PCR was performed using SYBR Green Master (Roche Diagnostics GmbH, Mannheim, Germany) in the LightCycler® 480 system (Roche Diagnostics GmbH). The primer sequences are listed in Table 2. β-actin was used as housekeeping control.

**Table 2:** Primers and their sequences for real time RT-PCR

Genes	Forward	Reverse
βactin	GGCACCACACCTTCTACAATG	GGGGTGTTGAAGGTCTCAAAC
TNF-α	TCTCATGCACCACCATCAAGGACT	ACCACTCTCCCTTTGCAGAACTCA
iNOS	TCTTTGACGCTCGGAACTGTAGCA	ACCTGATGTTGCCATTGTTGGTGG
IL-1β	AAGGGCTGCTTCCAAACCTTTGAC	ATACTGCCTGCCTGAAGCTCTTGT
IL-6	ATCCAGTTGCCTTCTTGGGACTGA	TAAGCCTCCGACTTGTGAAGTGGT
IL-10	GGCAGAGAACCATGGCCCAGAA	AATCGATGACAGCGCCTCAGCC
IL-12p40	ATGGCCATGTGGGAGCTGGAGAAAG	GTGGAGCAGCAGATGTGAGTGGCT
IL-23p19	ATGTGCCCCGTATCCAGTGT	GGGGTGATCCTCTGGCTGGA
ARG1	TTATCGAGCGCCTTTCTCAA	TGGTCTCTCAGGTCATACTCTGT
CD206	TCAGCTATTGGACGCGAGGCA	TCCGGGTTGCAAGTTGCCGT
CCL2	GCATCCACGTGTTGGCTCA	CTCCAGCCTACTCATTGGGATCA
CCL5	ACTCCCTGCTGCTTTGCCTAC	GAGGTTCCTTCGAGTGACA
CXCL2	AAGTTTGCCTTGACCCTGAA	AGGCACATCAGGTACGATCC
C1Qb	ATAAAGGGGGAGAAAGGGCT	CGTTGCGTGGCTCATAGTT
CD16	CAGAATGCACACTCTGGAAGC	GGGTCCCTTCGCACATCAG
CD32	AGGGCCTCCATCTGGACTG	GTGGTTCTGGTAATCATGCTCTG
CD64	AGGTTCCTCAATGCCAAGTGA	GCGACCTCCGAATCTGAAGA
CD36	ATGGGCTGTGATCGGAACTG	GTCTTCCCAATAAGCATGTCTCC
CD47	CACAGTCATCGTGGTTGTTGGA	GTGATCAATATGGCAATGGTGAAAG
Trem2	CTGGAACCGTCACCATCACTC	CGAAACTCGATGACTCCTCGG
Anxa1	ATGTATCCTCGGATGTTGCTGC	TGAGCATTGGTCCTCTTGGTA

### Luminex multiplex assay

The cytokine & chemokine magnetic bead panel kit (LXSAMSM-10, R&D, USA) was designed to detect CCL2, CCL5, CXCL2, CXCL10, Granulocyte-macrophage colony-stimulating factor (GM-CSF), IL-1β, IL-6, IL-10, IL-12p70 and TNF-α in the supernatant after different treatments. All procedures were carried out according to the manufacturer’s instructions. The signals were detected and data analyzed by the Bio-plex 200 System (BIO-RAD, Richmond, CA). Chemokine concentrations were calculated using StarStation software (Applied Cytometry Systems, UK) with a four parameter curve-fitting algorithm applied for standard curve calculation.

### Retinal explants and microglia co-culture

The method for retinal explants and microglia co-culture was adapted from a previously published paper [44]. Briefly, microglial cells from *Socs3*^*fl/fl*^ and DKO mice were seeded onto polycarbonate filters (Fisher scientific, UK) and cultured in DMEM/F12 for 24 h to allow firm attachment to the filters. Cells were then given M0/ M1/ M2 treatments for overnight. Cells were washed with PBS three times to remove stimuli. Retinas from C57BL/6 J mice were placed with the photoreceptor side in contact with microglia for 24 h at 37 °C. Retinal explants were fixed and stained for cone arrestin.

### Statistical analysis

Statistical analysis was performed in GraphPad Prism (GraphPad Software, San Diego, CA, USA) and SPSS. Student’s t tests were performed for analysis and comparisons between two groups. Two-way analysis of variance (ANOVA) followed by Sidak’s or Tukey multiple comparison test were used to investigate the difference between groups as indicated in individual figure legends. Data are presented as mean ± SD and significance was established as *P* < 0.05.

## Results

### General phenotype of the DKO mice

The DKO mice bred normally and litter size was similar to C57BL/6 WT and *Socs3*^*fl/fl*^ control mice. They were not prone to infections in our Specific-Pathogen-Free (SPF) standard housing facility. Some mice started showing signs of slower and limited movements at 7 ~ 8 months old. 57% of the DKO mice died before 12 months old and the majority of death occurred between 7 and 9 months of age (Additional file 3). Post-mortem investigations (*n* = 27 mice) showed that ~ 30% of the mice had tumors in the liver, spleen, and intestinal system. No visible abnormality was observed in the brain, the lung or the heart. The majority of the DKO mice died before 15 months of age. Our results suggest that the DKO mice may have accelerated aging.

### Retinal microglial activation in DKO mice

As the two immune regulatory genes, *Cx3cr1* and *Socs3,* were depleted exclusively in microglia in the retina, we examined microglial cells under normal aging conditions. For this purpose, we compared the DKO mice with each single KO mice (*Cx3cr1*^gfp/gfp^ and *LysMCre-Socs3*^*fl/fl*^) and used the *Socs3*^*fl/fl*^ mice as controls. Due to the short lifespan of the DKO mice, “aged” mice were 10–12 months old and “young” mice were 3–5 months old in this study.

**Fig. 1:**
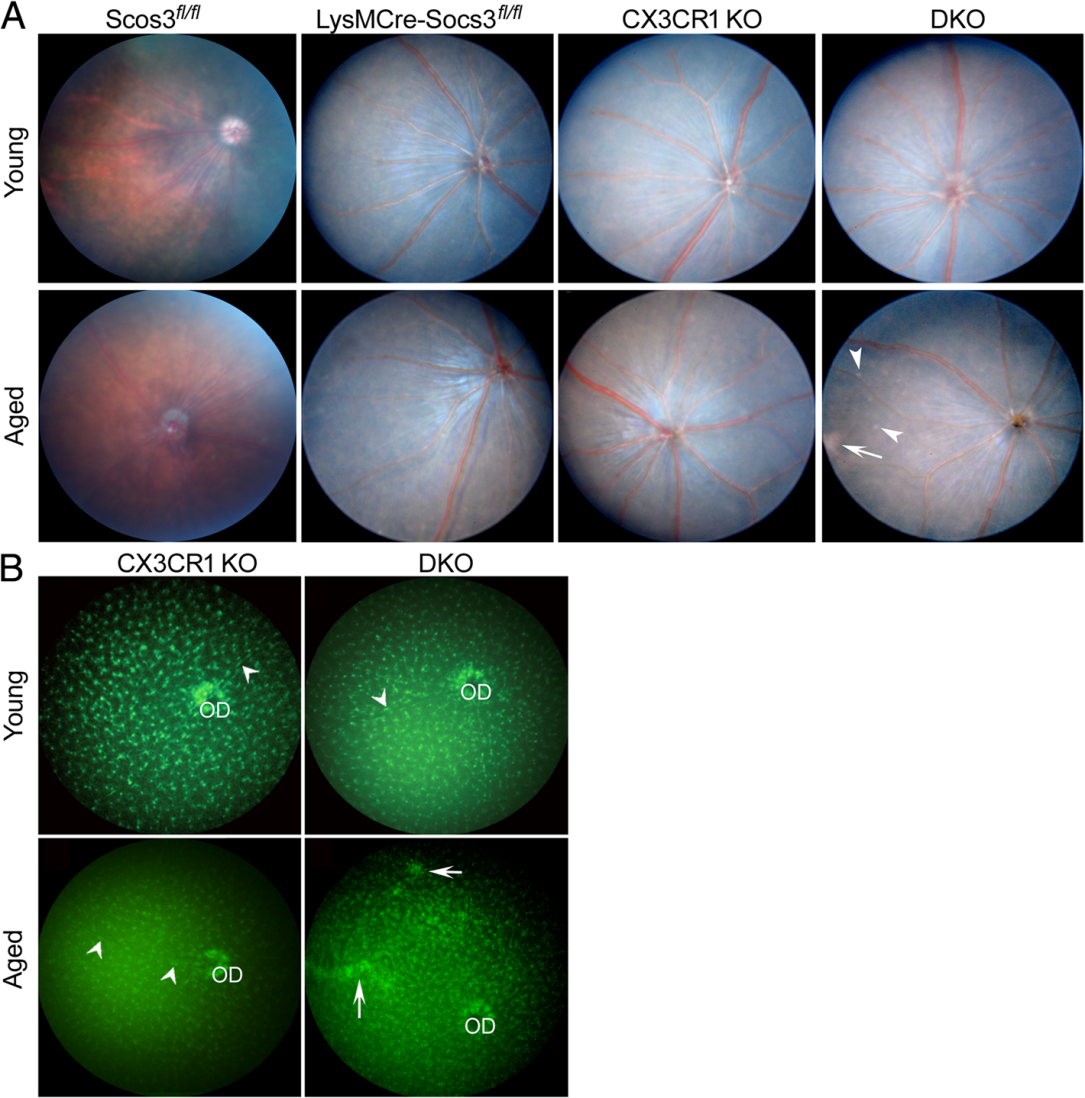
Fundus images and green fluorescence images of different mice. **a** Fundus images were taken using the topic endoscopic fundus imaging (TEFI) system from young (3-month) and aged (12-month old) *Socs3*^*fl/fl*^ *mice, LysMCre-Socs3*^*fl/fl*^ *mice, Cx3cr1*^*gfp/gfp*^ (CX3CR1 KO) mice, and DKO mice. Arrow – a patch of whitish lesion; arrowheads – multiple whitish dots. **b** Fundus green fluorescence images were taken from 3-month or 12-month old *Cx3cr1*^*gfp/gfp*^ and DKO mice using Micron IV. Arrows – patches of GFP aggregations; arrowheads – perivascular macrophages. OD – optic disc

Fundus images did not show any abnormality in 3 and 12 month-old *Socs3*^*fl/fl*^ mice, *LysMCre-Socs3*^*fl/fl*^ mice, and *Cx3cr1*^*gfp/gfp*^ mice, as well as 3 month-old DKO mice (Figs. 1a). Patches of whitish lesions and multiple small whitish dots were observed in 75% of 12 month-old DKO mice (arrow and arrowheads, Fig. 1a). These lesions were not observed in age-matched WT (*Socs3*^*fl/fl*^) and single KO mice (i.e., *LysMCre-Socs3*^*fl/fl*^, and *Cx3cr1*^*gfp/gfp*^).

Retinal microglia in the *Cx3cr1*^*gfp/gfp*^ and DKO mice were imaged in vivo due to their GFP expression. Dense GFP signals were observed in the optic disc (OD) in *Cx3cr1*^*gfp/gfp*^ and DKO mice (Fig. 1b). GFP^+^ microglia were evenly distributed throughout the fundus. Perivascular macrophages were also visible in some areas (Fig. 1b, arrowhead). No obvious difference was observed between 3 month old *Cx3cr1*^*gfp/gfp*^ and DKO mice. The density of GFP^+^ cells appeared to be increased in 12 month old DKO mice and patches of GFP aggregations were observed in these mice (Fig. 1b, arrows).

**Fig. 2:**
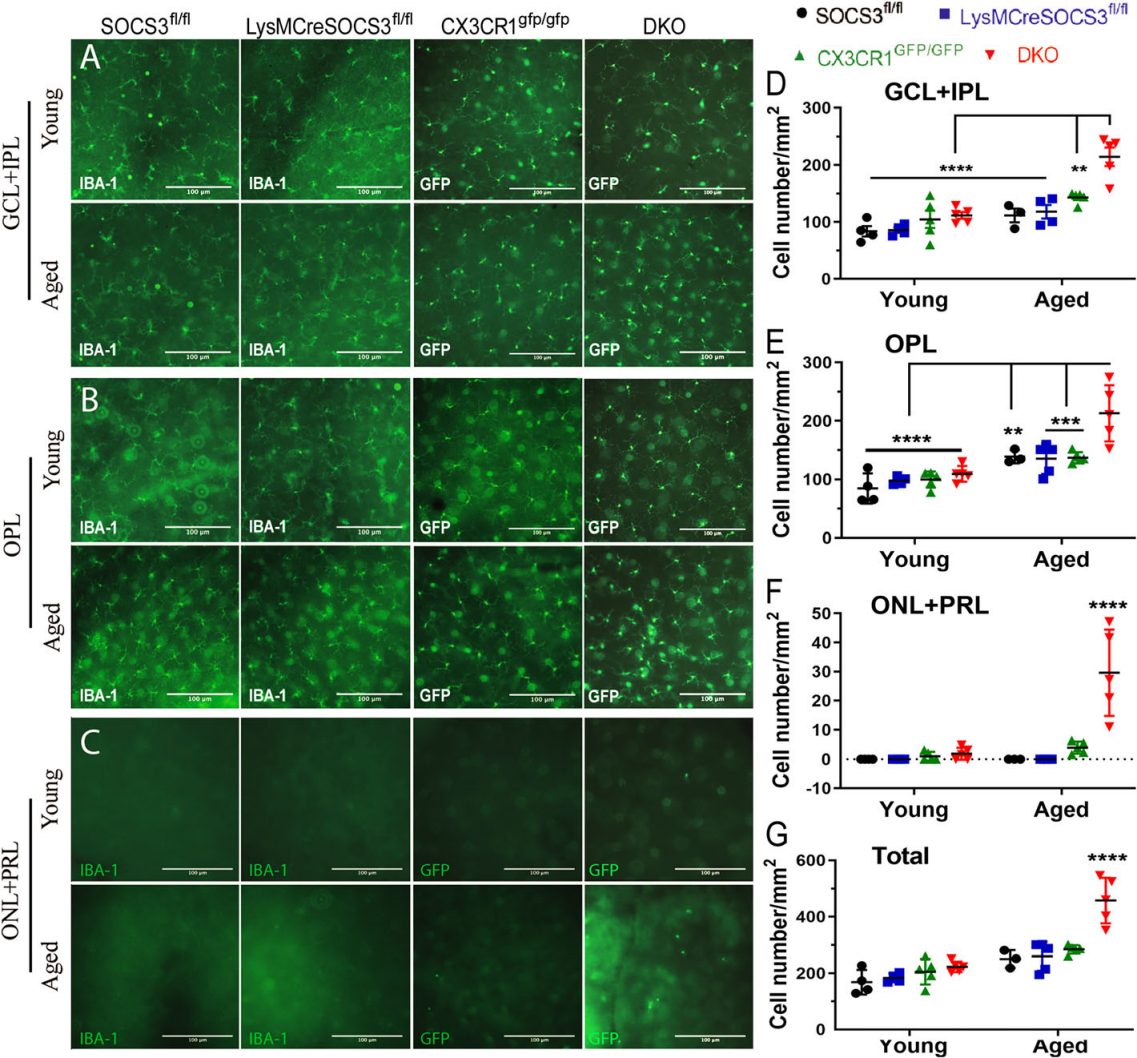
Retinal microglia in different groups of mice. Retinal flatmounts from young (3–5 months) and aged (10–12 months) *Socs3*^*fl/fl*^ and *LysMCre-Socs3*^*fl/fl*^ mice were stained for IBA-1. Retinal flatmounts from *Cx3cr1*^*gfp/gfp*^ and DKO of the same age remain unstained. All samples were imaged by Dmi8 fluorescence microscopy. **a**-**c** Representative images of retinal microglia from GCL + IPL (**a**), OPL (**b**) and ONL + PRL (**c**). **d**-**f** quantification of microglia in different retinal layers of different mice. (**g**) The total number microglia in all retinal layers. Mean ± SD, *N* = 3 ~ 5 mice, Scale bar: 100 μm. Two-way ANOVA, Tukey’s test, ** *P* < 0.01, *** *P* < 0.001, **** *P* < 0.0001 (**** showed the difference between aged DKO and other groups in **f** and **g**). GCL – ganglion cell layer; IPL – inner plexiform layer; OPL – outer plexiform layer; ONL – outer nuclear layer; PRL – photoreceptor inner/outer segment layer

Microglia were further quantified in retinal flatmounts in all groups. Microglia in *Socs3*^*fl/fl*^ and *LysMCre-Socs3*^*fl/fl*^ mice (which do not express GFP) were identified by IBA-1 staining. In 3 month old mice, IBA-1^+^ cells resided in the GCL and IPL (Fig. 2a, d), and OPL (Fig. 2b, e). The numbers of microglia in the GCL and IPL were comparable in all four groups; whereas DKO mice had higher number of microglia in the OPL than those in other strains (Fig. 2e). In the aged mice, in addition to GCL, IPL and OPL, microglia were detectable in the outer retinal layers, including ONL + PRL, particularly in DKO mice (Fig. 2c, f). Aged DKO mice had a significantly higher number of microglia than other age-matched strains in all layers studied (Fig. 2g). The number of microglia in RPE flatmounts of aged DKO mice were significantly higher than those in other age-matched strains (Additional file 4A, B). Microglia in photoreceptor layer and RPE flatmounts of aged DKO mice also expressed Isolectin B4, indicative of microglial activation (Additional file 4C). Our results suggest that age-related microglial activation was significantly enhanced in the DKO mice compared with *Socs3*^*fl/fl*^ and single KO mice.

### Retinal neuronal degeneration in aged DKO mice

To understand if uncontrolled microglial activation led to retinal pathology in aged DKO mice, we conducted ERG in these mice. Our result show that a-wave, b-wave and the OPs were significantly reduced in aged DKO mice compared with those in young DKO mice (Additional file 5), suggesting a global reduction in retinal neuronal function. Therefore, we conducted further immunohistological investigations on retinal neurons.

**Fig. 3:**
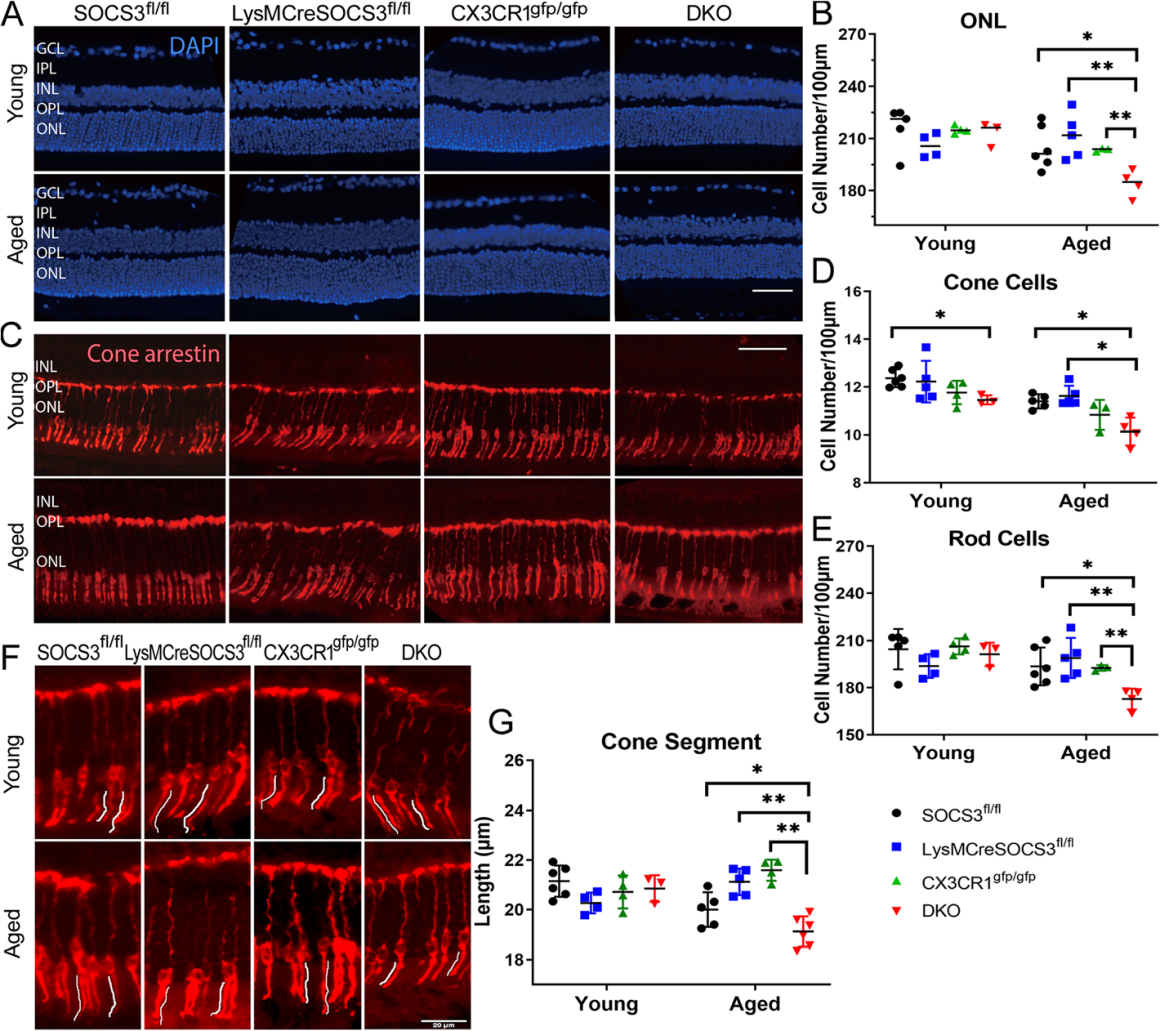
Photoreceptor alterations in different strains of young and aged mice. Retinal sections from young (3-5 m) and aged (10-12 m) *Socs3*^*fl/fl*^*, LysMCre-Socs3*^*fl/fl*^, *Cx3cr1*^*gfp/gfp*^ and DKO mice were stained with DAPI (**a**) or cone arrestin (**c**, **f**) and imaged by Dmi8 fluorescence microscopy. **a** Representative images showing DAPI stained of retinal sections from different groups of mice. **b** Quantitative analysis of DAPI^+^ cells in ONL in different groups of mice. **c** Representative images of cone arrestin^+^ cells in retinal sections in different groups of mice. **d** Quantitative analysis of cone arrestin^+^ cells in different groups of mice. **e** The number of rod cells in different groups calculated by deduction of cone arrestin^+^ cells from the total number of cells (DAPI^+^ cells) in the ONL. **f** High magnification images showing inner and outer segments of cone arrestin^+^ cells in different groups of mice. **g** Quantification of the length of cone photoreceptor segments. Mean ± SD; N = 3 ~ 6 mice. * *P* < 0.05, ** *P* < 0.01, Two-way ANOVA followed by Sidak’s multiple comparisons. Scale bar: 50 μm (**a**, **c**) or 20 μm (**f**). GCL – ganglion cell layer; IPL – inner plexiform layer; INL – inner nuclear layer; OPL – outer plexiform layer; ONL – outer nuclear layer

In young mice, with the exception of a slightly decreased number of cone photoreceptors in DKO mice, the numbers of cells in the entire ONL, cone arrestin^+^ photoreceptors, and rod photoreceptors were comparable in different strains of mice (Fig. 3a-e). In aged mice, the numbers of cells in the ONL, cone arrestin^+^ photoreceptors and rod photoreceptors were significantly decreased in DKO mice compared with single KO and *Socs3*^*fl/fl*^ mice (Fig. 3a-e), accompanied by reduction of the length of cone photoreceptor segments (Fig. 3f-g).

**Fig. 4:**
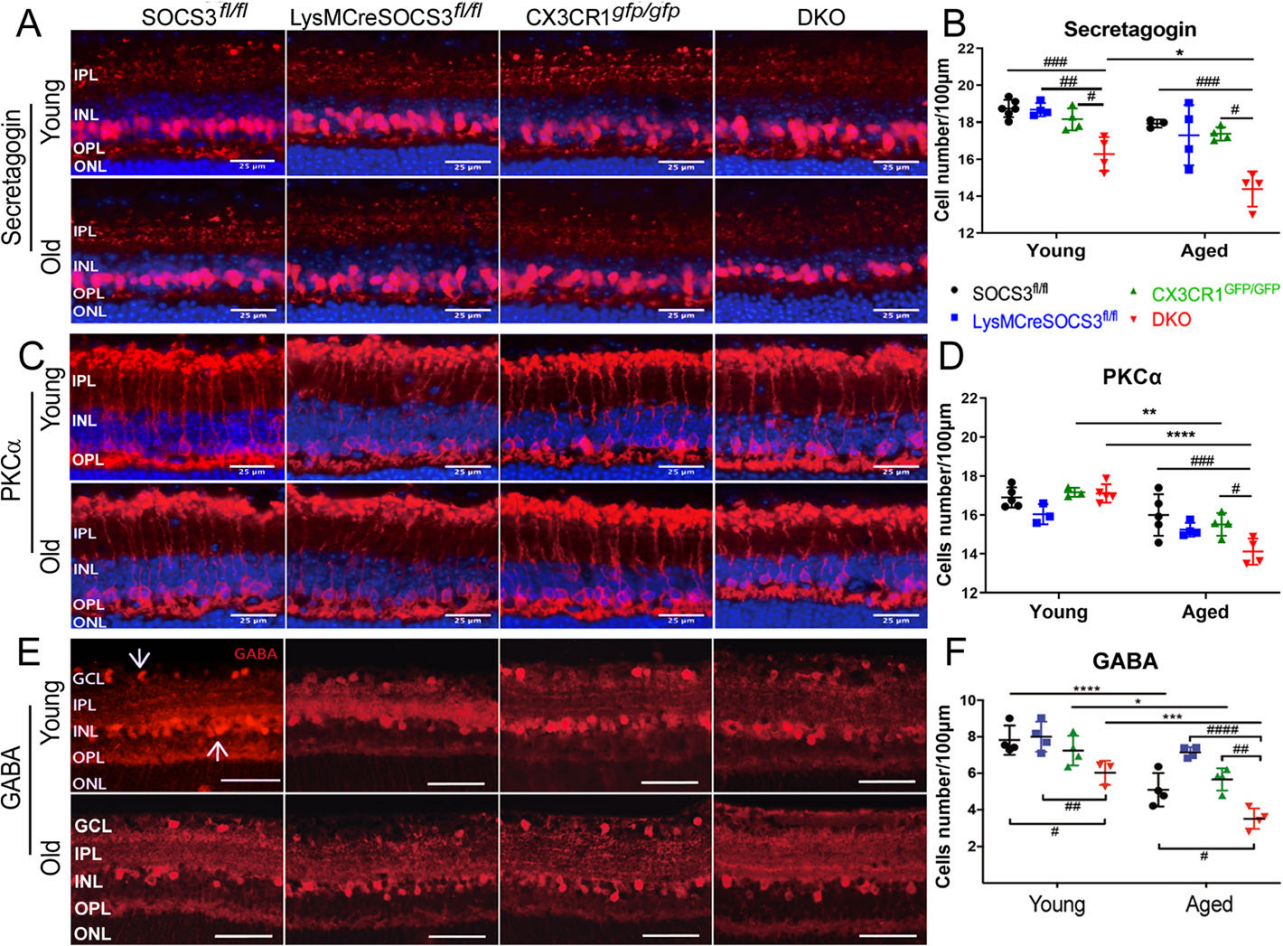
Retinal neurons in different strains of young and aged mice. Retinal sections from young (3-5 m) and aged (10-12 m) *Socs3*^*fl/fl*^*, LysMCre-Socs3*^*fl/fl*^, *Cx3cr1*^*gfp/gfp*^ and DKO mice were stained with secretagogin (**a**), PKCα (**c**) and GABA (**e**) and imaged by Dmi8 fluorescence microscopy. Sections in (**a**) and (**c**) were count-stained for DAPI. Quantitative analysis of secretagogin^+^ (**b**) and PKCα^+^ cells (**d**). (**f**) Quantitative analysis of GABAergic cells in both GCL and INL (white arrows in **e**). Scale bar: 25 μm. Mean ± SD, N = 3 ~ 5 mice, * *P* < 0.05, ** *P* < 0.01, *** *P* < 0.001, **** *P* < 0.0001; Two-way ANOVA followed by Tukey’s test. GCL – ganglion cell layer; IPL – inner plexiform layer; INL – inner nuclear layer; OPL – outer plexiform layer; ONL – outer nuclear layer

The number of secretagogin^+^ rod-bipolar cells was lower in young DKO mice than age-matched single KO or *Socs3*^*fl/fl*^ controls and was further reduced in aged DKO mice (Fig. 4a-b). The number of PKCα^+^ cone-bipolar cells was comparable in young mice (Fig. 4c-d). An age-related reduction was observed in *Cx3cr1*^*gfp/gfp*^ and DKO mice although the reduction was more significant in aged DKO mice (Fig. 4c-d). The number of GABAergic^+^ cells (including GABA^+^ amacrine cells in the INL and displaced amacrine cells in the GCL) was significantly lower in young DKO mice (Fig. 4e-f). The age-related reduction was observed in all strains of mice although the reduction was more profound in DKO mice (Fig. 4e-f).

**Fig. 5:**
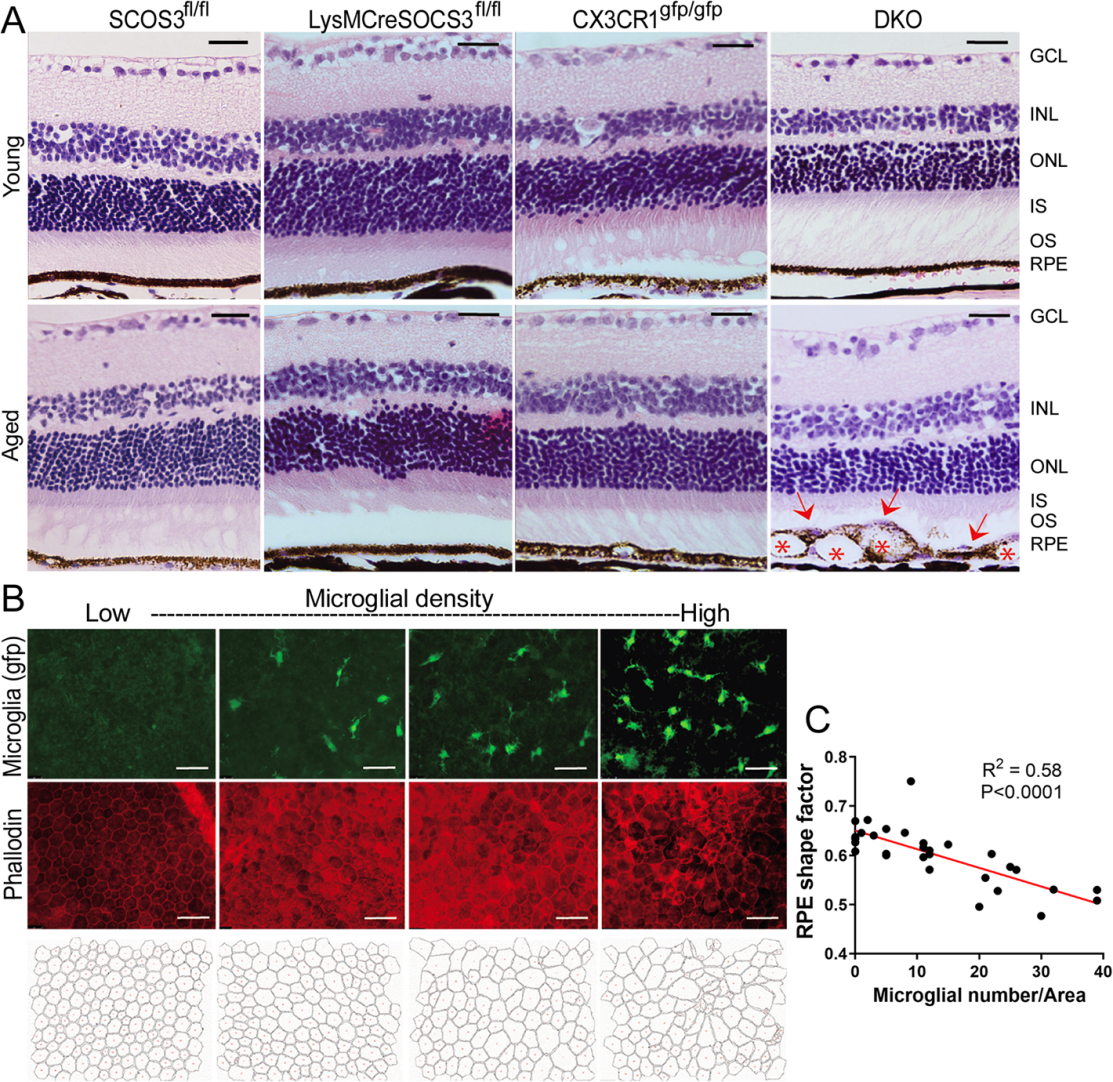
Retinal pigment epithelial cell alterations in different strains of young and aged mice. **a** Eyes from young (3-5 m) and aged (10-12 m) *Socs3*^*fl/fl*^*, LysMCre-Socs3*^*fl/fl*^*, Cx3cr1*^*gfp/gfp*^ and DKO mice were embedded in paraffin and retinal sections were subjected to haematoxylin and eosin (H&E) staining. Images were taken from the central and mid-peripheral area of the retina. Asterisks – vacuoles inside RPE; Red arrows – Purple stained materials (possibly cell nuclei) on top of RPE. Scale bar: 50 μm. GCL – ganglion cell layer; INL – inner nuclear layer; ONL – outer nuclear layer; IS - inner segments of photoreceptors; OS - outer segments of photoreceptors; RPE – retinal pigment epithelium. **b** RPE/choroidal flatmounts from 10 to 12 months old DKO mice were stained for phalloidin (red) and imaged by Dmi8. GFP^+^ infiltrating subretinal cells (macrophages or microglia) were in green. Representative images showing areas of normal RPE morphology with no infiltrating cells and different grades of RPE dysmorphologies with different numbers of infiltrating GFP^+^ cells. The outlines of individual RPEs were sketched at the bottom using FIJI. Scale bar: 50 μm. **c** Correlation between RPE shape factor and the number of infiltrating GFP cells. Statistics were applied by five images per mouse from six aged DKO mice

Histological investigation did not show any structural abnormalities in eyes from young mice (Fig. 5a). Localized RPE swelling (Fig. 5a, arrows) and vacuoles (Fig. 5a, asterisks) with thinner ONL in corresponding area were frequently observed in aged DKO mice. RPE damage in aged DKO mice was further confirmed in flatmount investigations and the lesions were always accompanied by microglial/macrophage infiltration (Fig. 5b). Aged DKO mice had enlarged RPE cell size, increased perimeter and reduced cell density. Areas of total RPE damage with massive microglial infiltration were frequently observed in aged DKO mice (Fig. 5b). The number of infiltrating microglia/macrophages negatively correlated with RPE shape factor in aged DKO (Fig. 5c).

In line with retinal neuronal degeneration, we also observed increased GFAP expression in 10 ~ 12 month old DKO mice (Additional file 6), indicative of Müller gliosis.

Our results suggest that aged DKO mice suffered from significant neuroretinal degeneration and RPE damage.

### Enhanced phagocytic activity in microglia from DKO mice

**Fig. 6:**
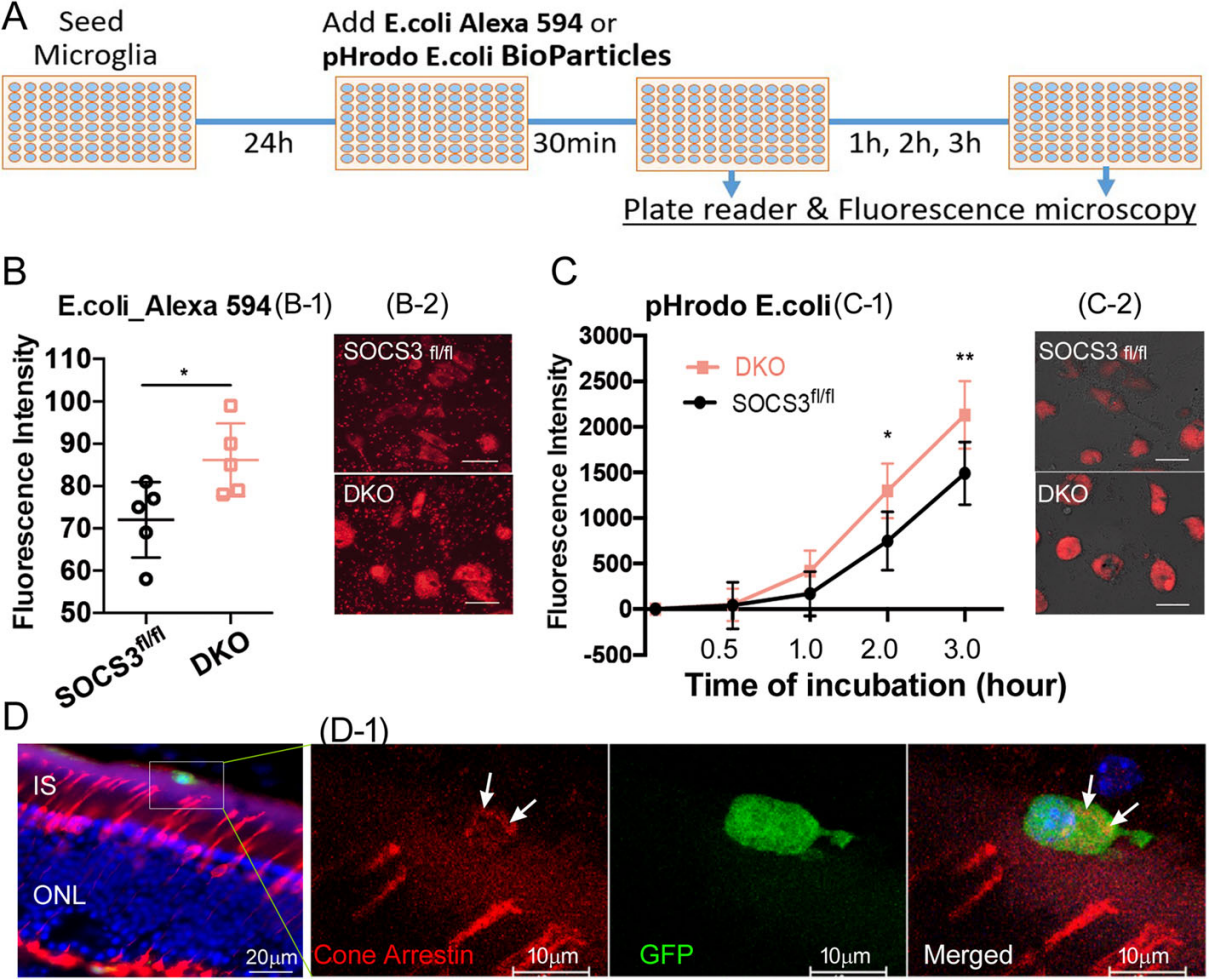
Phagocytic activity of microglia from *Socs3*^*fl/fl*^ and DKO mice. (A) Microglia from *Socs3*^*fl/fl*^ or DKO mice were seeded into 96-well plates. 24 h later, *E.coli* Alexa 594 Bioparticles or pHrodo *E.coli* Bioparticles were added into the wells. Fluorescence intensities were measured by plate reader and images were taken by fluorescence microscopy at the end of the study. (B) Fluorescence conjugated Alexa Fluor 594 *E.coli* particles were added to microglia for 30 min and the fluorescence intensities (B-1) were measured using Fluostar Omega microplate reader. After that, images were taken by Dmi8 fluorescence microscopy (B-2). (C) The pH sensitive *E.coli* particles were added to microglia. Fluorescent intensities (C-1) were measured using the Fluostar Omega microplate reader 0.5 h, 1 h, 2 h, and 3 h later. At the end of the study, images (C-2) were taken using Dmi8. Mean ± SD, *N* = 5 (B) and 3 (C). * *P* < 0.05, ** *P* < 0.01. Independent sample t test (B); Two-way ANOVA followed by Turkey’s test (C). Scale bar: 10 μm. (D) A confocal image from an aged DKO eye section showing cone arrestin (red) and GFP^+^ subretinal microglia (green). (D-1) Zoomed view of the marked area in (D) showing cone arrestin antigens (white arrows) inside a GFP^+^ filtrating cell. IS – inner segments of photoreceptors; ONL – outer nuclear layer

To understand how *Cx3cr1* and *Socs3* depletion may affect microglial function, microglial cultures from the DKO and *Socs3*^*fl/fl*^ mice were subjected to phagocytosis assay using the Alexa Fluor™ 594 conjugate *E. coli* BioParticles™ and the pH-sensitive pHrodo™ Red *E. coli* BioParticles™ systems. In both assays, microglia from DKO mice had significantly higher levels of fluorescence intensities compared to cells from *Socs3*^*fl/fl*^ control mice (Fig. 6A-C). Fluorescence microscopy further showed intense intracellular particles in DKO cells treated with Alexa Fluor™ 594 *E. coli* bioparticles (although un-ingested extracellular bioparticles were also visible) (Fig. 6B-2). In cells treated with pH-sensitive bioparticles, fluorescent signals were inside the cells and DKO cells demonstrated brighter fluorescent signals than *Socs3*^*fl/fl*^ cells (Fig. 6C-2). Our results suggest that the phagocytic activity of DKO microglia was significantly higher than that of *Socs3*^*fl/fl*^ cells. Interestingly, we also detected cone arrestin antigens inside GFP^+^ cells in the outer layers of retina from aged DKO mice but not in other strains (Fig. 6D), an indication of in situ uptake of damaged cone photoreceptors by microglia.

**Fig. 7:**
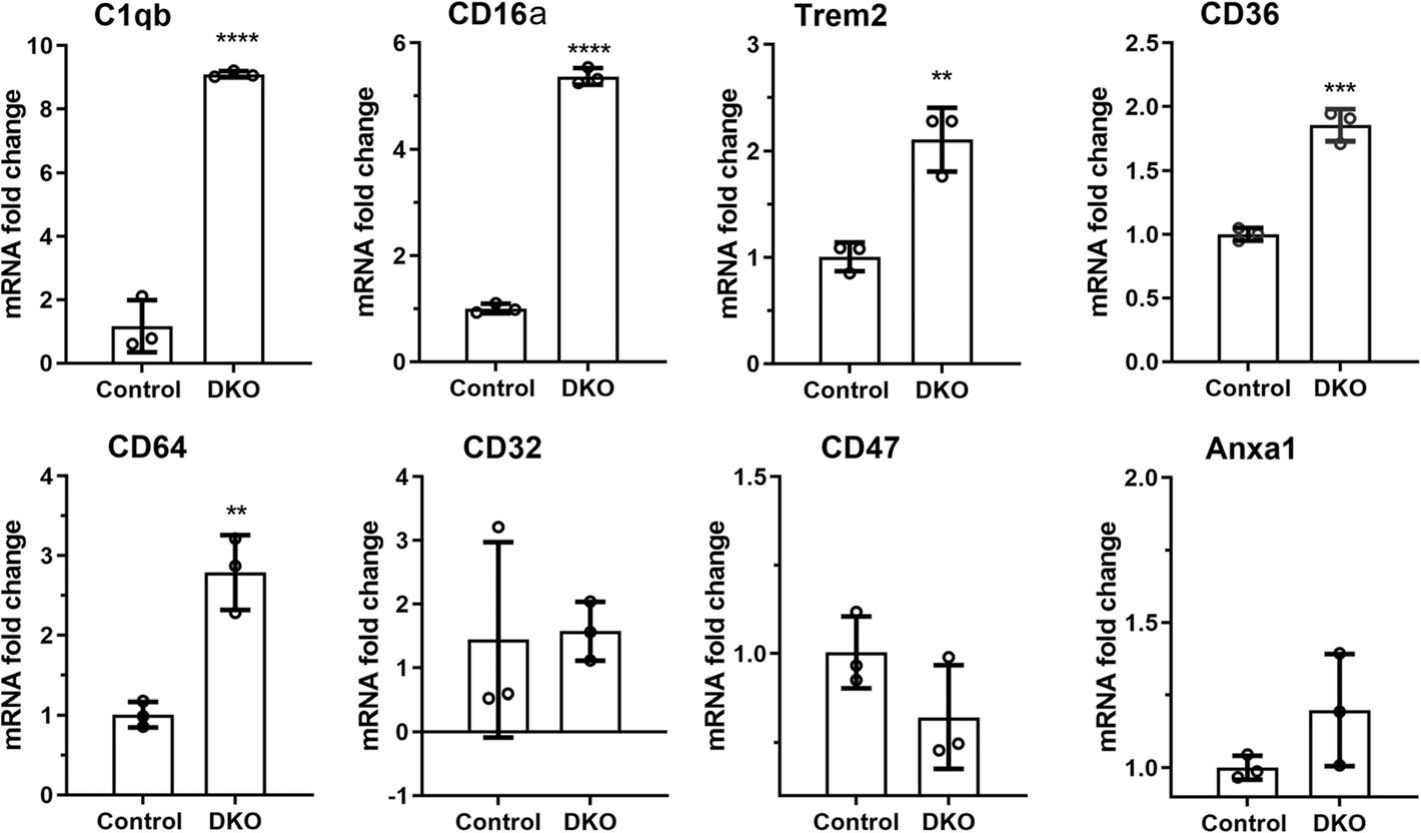
The mRNA expression of phagocytosis related genes in microglia from *Socs3*^*fl/fl*^ and DKO mice. The mRNA was extracted from brain microglia cultured from *Socs3*^*fl/fl*^ and DKO mice. The expression of C1qb, CD16a (Fcgr3), Trem2, CD36, and CD64 (Fcgr1), CD32 (Fcgr2b), CD47, and Anxa1was evaluated by qRT-PCR. Mean ± SD, *N* = 3. ** *P* < 0.01; **** *P* < 0.001, unpaired Student t test

Real-time RT-PCR showed that microglia from DKO mice expressed significantly higher levels of C1qb, CD16 (Fcgr3), Trem2, CD36, and CD64 (Fcgr1) compared to microglia from *Socs3*^*fl/fl*^ mice (Fig. 7). The expression of CD32 (Fcgr2b), CD47, and Anxa1 did not differ between DKO and *SOCS3*^*fl/fl*^ microglia (Fig. 7). The higher phagocytic activity of DKO microglia may be related to higher levels of C1qb, Trem2, CD36, and Fc-gamma receptor expression by these cells.

### Altered cytokine expression and secretion by microglia from DKO mice

**Fig. 8:**
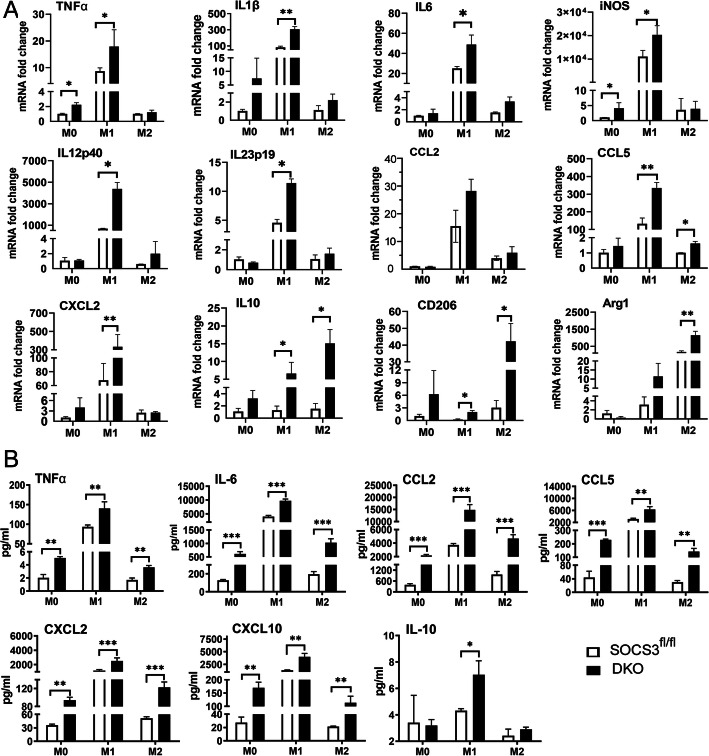
The expression and production of cytokines and chemokines by microglia from *Socs3*^*fl/fl*^ and DKO mice. Brain microglia from *Socs3*^*fl/fl*^ and DKO mice were untreated (M0, naive microglia) or treated with LPS + IFN-γ (M1) or IL-4 (M2). The mRNA expression of TNF-α, IL-1β, IL-6, iNOS, IL-12p40, IL-23p19, CCL2, CCL5, CXCL2, IL-10, CD206 and Arg1 were examined by real-time RT-PCR (**a**). The protein levels of TNF-α, IL-6, CCL2, CCL5, CXCL2, CXCL10 and IL-10 in the supernatants were measured using the cytokine Luminex multiple assay (**b**). Mean ± SD, N = 3. * *P* < 0.05; ** *P* < 0.01; *** *P* < 0.001. Two-way ANOVA followed by Tukey’s multiple comparisons. Experiments were repeated three times

Under normal culture conditions, naïve microglia (M0) from DKO mice expressed significantly higher levels of TNF-α and iNOS mRNA (Fig. 8a) and produced significantly higher amounts of TNF-α, IL-6, CCL2, CCL5, CXCL2 and CXCL10 proteins compared to their counterparts from *Socs3*^*fl/fl*^ mice (Fig. 8b).

Following LPS + IFN-γ stimulation (M1), increased mRNA expressions of pro-inflammatory mediators such as TNF-α, iNOS, IL-1β, IL-6, IL-12p40, IL-23p19, CCL2, CCL5 and CXCL2 were observed in microglia from both *Socs3*^*fl/fl*^ and DKO mice compared to naïve M0 cells although the upregulations were generally more significant in DKO M1 cells compared to *Socs3*^*fl/fl*^ M1 cells (Fig. 8a). M1 microglia from DKO mice produced and released significantly higher levels of TNF-α, IL-6, CCL2, CCL5, CXCL2, CXCL10 and IL-10 compared with M1 microglia from WT mice (Fig. 8b). Interestingly, although the mRNA of IL-1β was significantly increased in M1 microglia, this cytokine was below detectable levels in the supernatants (data not shown).

IL-4 treated microglia (M2) from DKO mice expressed significantly higher levels of IL10, CD206 and Arg1 mRNA compared to their counterpart from *Socs3*^*fl/fl*^ mice (Fig. 8a). Interestingly, the productions of TNF-α, IL-6, CCL2, CCL5, CXCL2 and CXCL10 were also significantly higher in IL-4-treated DKO microglia than those in IL-4-treated *Socs3*^*fl/fl*^ microglia (Fig. 8b).

Together, our data suggested that microglia with *Socs3* and *Cx3cr1* deficiency produce significantly higher basal levels of cytokines and chemokines, and they are also more sensitive to cytokine stimuli (such as LPS + IFNγ or IL-4).

### Microglia induced photoreceptor death in retinal explants

**Fig. 9:**
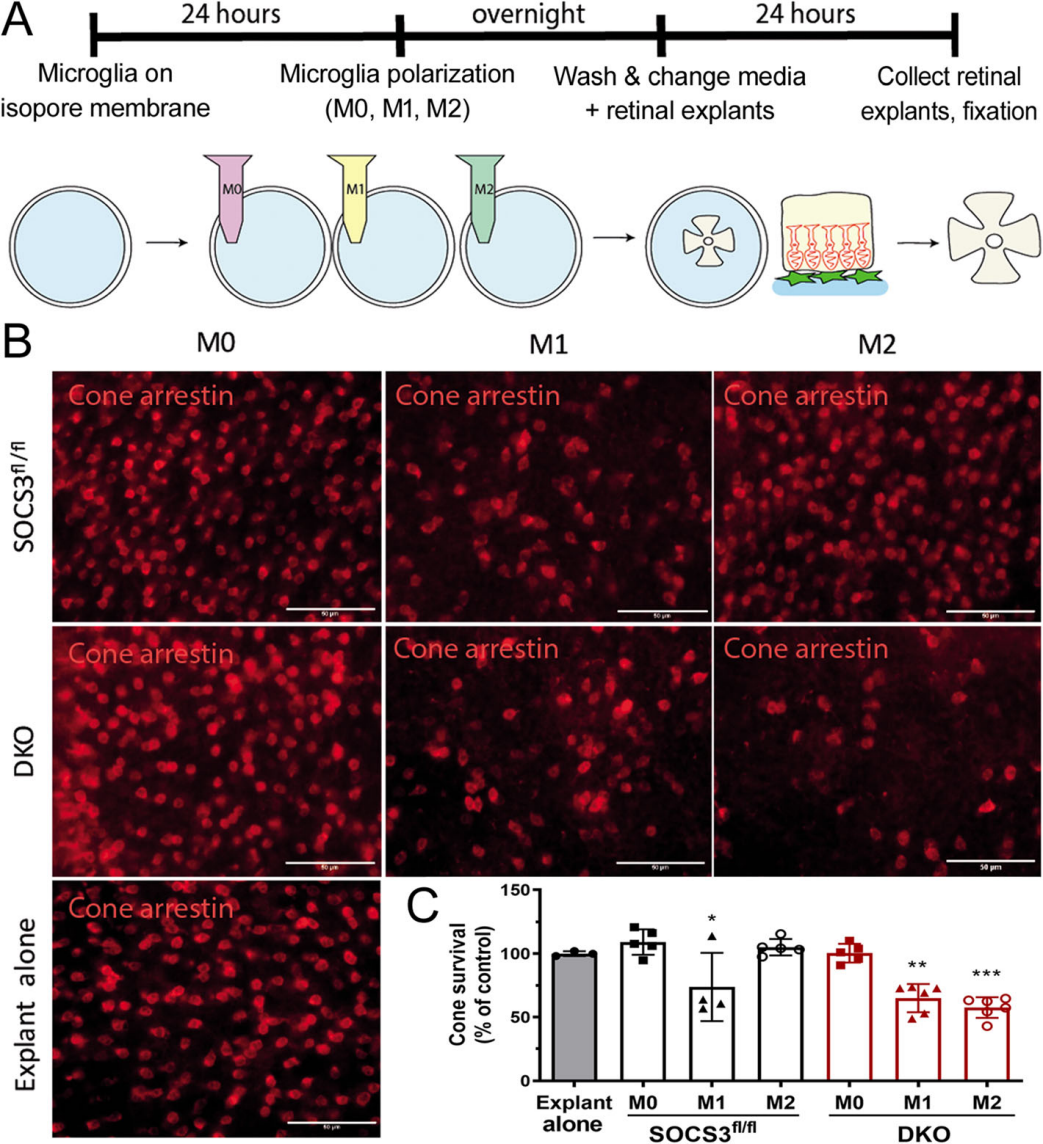
The effect of microglia from *Socs3*^*fl/fl*^ and DKO mice on photoreceptor survival in retinal explant cultures. Brain microglia were cultured from *Socs3*^*fl/fl*^ and DKO mice. Retinal explants were from *Socs3*^*fl/fl*^ mice. Retinal explants were co-cultured with different types of microglia for 24 h. The flatmounts of retinal explants were then stained for cone arrestin and imaged by Dmi8 fluorescence microscopy. **a** Schematic diagram showing study design. **b** Representative images showing cone arrestin^+^ cells in retinal explants in different treatment groups. **c** Quantitative data of cone arrestin^+^ cells in different groups. The number of cone arrestin^+^ cells in different treatment groups was compared to retinal explants without microglia co-culture. Mean ± SD. N = 3 ~ 6 explants. * *P* < 0.05, ** *P* < 0.01, *** *P* < 0.001. Two-way ANOVA followed by Tukey’s multiple comparisons. Scale bar: 50 μm

To further understand if altered microglial activation contributes to retinal degeneration in aged DKO mice, retinal explants from C57BL/6 J mice were cultured alone or co-cultured with microglia from *Socs3*^*fl/fl*^ and DKO mice without and with M1 or M2 polarization (Fig. 9a), and the number of cone arrestin^+^ cells was evaluated in retinal flatmounts. The addition of naïve (M0) microglia from *Socs3*^*fl/fl*^ or DKO mice did not affect the number of cone arrestin^+^ cells compared to retinal explants alone (109.14 and 100.42% respectively, Fig. 9b, c). M1-type microglia from both *Socs3*^*fl/fl*^ and DKO significantly reduced cone photoreceptor survival (73.67 and 64.87% respectively, Fig. 9b, c). Interestingly, M2-type microglia from DKO but not *Socs3*^*fl/fl*^ mice also significantly reduced cone photoreceptor survival (57.48%, Fig. 9b, c). Our results suggest that active microglia from DKO mice are toxic to photoreceptors regardless of how they are activated; whereas, microglia from *Socs3*^*fl/fl*^ mice are only toxic to photoreceptors when they are activated to a pro-inflammatory phenotype.

## Discussion

In this study, we showed that deletion of two immune negative regulators, *Cx3cr1* and *Socs3* (in LysM^+^ cells) resulted in uncontrolled retinal microglial activation during aging, accompanied by a significant degeneration of retinal neurons and RPE cells. Although *Cx3cr1* was deleted globally, it is expressed exclusively by microglia and perivascular macrophages in the retina. *Lysozyme 2* (*LysM* or *Lyz2*) is expressed by all retinal microglia and macrophages and by a small population (< 8%) of ganglion cells and amacrine cells [45]. Deletion of *Cx3cr1* and *Socs3* in the DKO mice would therefore predominately affect retinal microglia. The degeneration of retinal neurons in the aged DKO mice was likely the consequence of primary microglial dysfunction. Therefore, retinal pathologies observed in this study fall into the disease category of “microgliopathay” [46].

The DKO mice were not prone to any infection when housed in a standard SPF facility although the lifespan of the DKO mice was significantly shortened compared to WT or single KO mice. The DKO mice appeared to have slower and limited movements and higher incident of tumor development (evidence of accumulation of mutations). This suggests that the DKO mice might have accelerated aging. Therefore, this mouse model may be valuable for understanding the contribution of low-grade chronic inflammation (inflammaging) [47] to the aging process.

Retinal aging is accompanied by a low-grade of parainflammation characterized by mild microglial activation, subretinal migration and complement activation [33, 48], and a dysregulated parainflammatory response contributes critically to age-related retinal degenerative diseases such as AMD [11]. Previous studies have shown that the mice with *Cx3cr1* deficiency developed retinal degeneration by 1–2 years of age [49, 50], and the pathology is known to be related to increased subretinal infiltration of CCR2^+^ phagocytes and the impairment in their removal [50]. In our study, significant microglial activation and retinal degeneration were only observed in DKO mice by the age of 10–12 months. Our results suggest that microglial activation is collectively regulated by neuron-derived signals (e.g., CX3CL1) and intracellular immune inhibitors (e.g., SOCS3). The discrepancy in retinal pathology in *Cx3cr1* KO mice between our study and that of Combadiere et al. may be related to different housing conditions.

We observed a number of functional alterations in microglia from the DKO mice. These include higher cytotoxicity to photoreceptors in retinal explants, significantly higher levels of phagocytic activities and more secretory proteins (TNF-α, IL-6, CCL2 CCL5, CXCL2, CXCL10 and IL-10). The secretory profile was similar to the senescence-associated secretory phenotype (SASP) of macrophages [51] with the exception of IL1β, which was not detected in microglial supernatants in our study. The JAK/STAT pathway is known to be involved in cell senescence and the phenotype of SASP [52] and JAK inhibition alleviated SASP phenotype and frailty in old age [52]. Previously, we have shown that the *LysMCre-Socs3*^*fl/fl*^ mice had higher levels of pSTAT3 in the retina [53]. Furthermore, a recent study has shown that CX3CR1-deficient microglia exhibit a premature aging transcriptome [54]. It is possible that microglia from the DKO mice may also undergo premature aging although this warrants further investigation.

SASP can have significant pathological effects on the aging retina. In our study, microglia from DKO mice produced significantly higher levels of cytokines and chemokines compared to their counterparts regardless of their activation states (Fig. 8a); however, only M1 and M2 (but not naïve M0) microglia were toxic to photoreceptors in retinal explants (Fig. 9b-c). Compared to naïve (M0) microglia, M2 microglia from DKO mice produced higher levels of IL-6, CCL2 and CXCL2, suggesting that the three mediators might contribute to M2 microglia-mediated photoreceptor death.

The high phagocytic activity of DKO microglia may be due to the increased expression of relevant receptors such as C1q, Trem2, FcγR and CD36 (Fig. 7). The underlying mechanism related to the upregulation of these surface molecules remains unknown but may be related to uncontrolled activation of microglia in DKO mice. For example, C1q expression in microglia is known to be upregulated during brain injury [55] and infection [56]. Increased TREM2 expression was reported in amyloid plaque-associated microglia [57]. Altered expression of phagocytosis-related molecules and enhanced phagocytic activity appear to be an important part of primary microglial dysfunction in our model system.

A recent study has shown that microglial function is distinct in different anatomical locations during retinal homeostasis and degeneration [58]. We have shown that microglia in different retinal layers are regulated by diverse neurons and molecules [25]. Specifically, ganglion cell and amacrine cells work together to control microglial activation in the GCL/IPL layer; whereas microglia in the OPL is regulated by horizontal cells and photoreceptors assisted by bipolar cells [25]. In the present model of retinal microgliopathy, microglia in all retinal layers were activated and a wide range of retinal neurons and RPE cells were affected. The initial trigger of retinal microglial activation in the aging DKO mice would likely be oxidative stress. Previously, we detected ox-LDL and 2,4-Dinitrophenol (DNP) in all retinal layers of the aged mice [33] suggesting ubiquitous oxidative stress in the aging retina. Our results also suggest that that Cx3cr1 and Socs3 are involved in the parainflammatory response of microglia in all retinal layers.

The crosstalk between microglia and retinal neurons is important for maintaining retinal homeostasis. Active microglia release excessive amounts of proinflammatory cytokines and chemokines that kill retinal neurons. Dead neurons in turn, further stimulate microglial activation. The reduction in retinal neurons also weakens the inhibitory signals to microglia further escalating microglial activation. Apart from expressing the SASP, microglia in the DKO mice had enhanced phagocytic activities and the hyperactive engulfment (named as phagoptosis by [59]) can also lead to unnecessary neuron loss [60]. Recent evidence indicates that phagoptosis contributes to neuronal loss in various CNS diseases [61–63].

The damage to RPE cells in our model may be a consequence of excessive retinal neuronal degeneration. We observed a strong correlation between the number of subretinal microglia/macrophage and RPE dysmorphology (Fig. 6B-C). Damaged neurons are removed from the retina by subretinal phagocytes (including microglia), likely through the subretinal space – RPE – choroid route (a CD47-dependent pathway) [64]. Impaired elimination ability of these subretinal phagocytes is known to contribute to RPE damage and the development of age-related macular degeneration [65]. Although we do not know if the mechanisms involved in subretinal phagocyte elimination is altered in the DKO mice, the continued retinal neuronal damage during aging may lead to accumulation of active phagocytes into the subretinal space, which may cause RPE damage [65].

## Conclusions

Age-induced retinal microglial activation in the *LysMCre-Socs3*^*fl/fl*^*Cx3cr1*^*gfp/gfp*^ *D*KO mice is dysregulated. This uncontrolled low-grade microglial activation resulted in retinal microgliopathy characterized as extensive neuronal degeneration and RPE damage. In this model of microgliopathy, active microglia may damage neurons through SASP expression or phagoptosis although the underlying mechanisms remain to be investigated. This model offers a unique opportunity to study the mechanisms of microglial dysfunction-mediated retinal pathology and will also help to elucidate the detrimental role of inflammaging in age-related retinal degeneration.

## Supplementary Information

**Additional file 1. **Genotype of the DKO mice. Genotyping PCRs were performed using primer combinations distinguishing the WT and knockout alleles for *LysMCre, Socs3* and *Cx3cr1* respectively. (A) Primers used for mouse genotyping. (B) Representative PCR images. For *Socs3*^*fl/fl*^ loci, DKO mice showed the band at 700 bp while wild type (WT) sample showed band at 500 bp and the heterozygous showed both bands. For the *LysMCre* loci, both DKO and heterozygous samples showed band at 700 bp while the WT at 350 bp. DKO mice showed a band of 1200 bp for *Cx3cr1* allele, while WT showed band at 970 bp and the heterozygous showed both bands.

**Additional file 2.** Mouse strain used for each experiment.

**Additional file 3.** The death of DKO mice during aging. The life span of DKO mice was recorded during aging. 27 out of 47 mice died by the age of 12 months old. Data showing the percentage of mice died at different ages.

**Additional file 4. **Microglial sub-retinal accumulation and Isolectin B4 expression in different strains of young and aged mice. Microglia were labelled by immunostaining of Isolectin B4 (IBA4) in young (3-5m) and aged (10-12m) mice. (A) Representative images showed RPE/choroid flatmounts staining. Microglia are visualised by IBA-1 staining (green) in *Socs3*^*fl/fl*^ and *LysMCre-Socs3*^*fl/fl*^ mice or the gfp tag in *Cx3cr1*^*gfp/gfp*^ and DKO mice. All samples were imaged by Dmi8 fluorescence microscopy (see Materials and Methods). (B) The graph showed quantification of microglia in RPE/choroid flatmounts of different mice. (C) Activated microglia (GFP^+^) were detected by Isolectin B4 staining in aged DKO retina in both photoreceptor layer and RPE flatmount. Mean ± SD. N ≥ 3 mice, Scale bar: 1000 μm (A) and 50 μm (C). Two-way ANOVA, Tukey’s test, **, P < 0.01.

**Additional file 5. **Electroretinogram (ERG) in *LysMCre-Socs3*^*fl/fl*^*Cx3cr1*^*gfp/gfp*^ DKO mice. Ganzfeld ERG was conducted in young (3 months old) and aged (11 months old) DKO mice with different intensities of flash light. (A) a-wave amplitudes. (B) b-wave amplitudes. (C) Oscillatory potentials. Mean ± SD, n = 8, *P<0.05; **P<0.01; ***P<0.005; ****P<0.001. Two-way ANOVA with Sidak’s multiple comparison test in (A, B). Unpaired Student t test was used in C.

**Additional file 6. **GFAP expression in different strains of young and aged mouse retina. Retinal sections from young (3-5m) and aged (10-12m) *Socs3*^*fl/fl*^*, LysMCre-Socs3*^*fl/fl*^*, Cx3cr1*^*gfp/gfp*^ and DKO mice were stained with GFAP and DAPI to assess Müller glia activation. (A) Representative images showing GFAP stained of retinal sections from different groups of mice. Scale bar: 50 μm. (B) Quantitative analysis of GFAP^+^ area in different groups of mice. Two-way ANOVA followed by Sidak’s multiple comparisons. N ≥ 3 mice, *, P < 0.05 compared young and aged DKO; ^#^, P<0.05 between old DKO and old *Socs3*^*fl/fl*^.

## Data Availability

All data generated or analysed during this study are included in this published article (and its additional files).

## Abbreviations

AMD: Age-related macular degeneration
Arg1: Arginase 1
CCL: C-C Motif Chemokine Ligand
CD: Cluster of differentiation
CNS: Central nervous system
CXCL: Chemokine (C-X-C motif) ligand
DAMPs: Damage-associated molecular patterns
DKO: Double knock-out
GCL: Ganglion cell layer
GFP: Green fluorescent protein
IB4: The α-d-galactosyl-specific isolectin B
IBA-1: Ionized calcium binding adaptor molecule 1
IFN-γ: Interferon gamma
IL: Interleukin
IPL: Inner plexiform layer
JAKs: Janus kinases
KO: Knock-out
LPS: Lipopolysaccharide
NLRs: NOD-like receptors
ONL: Outer nuclear layer
OPL: Outer plexiform layer
PAMPs: Pathogen-associated molecular patterns
PRL: Photoreceptor layer
PRRs: Pattern recognition receptors
RLRs: RIG-like receptors
RPE: Retinal pigment epithelium
RT-PCR: Real time-polymerase chain reaction
Socs3: Suppressor of cytokine signaling 3
STATs: Signal transducer and activator of transcription proteins
TLRs: Toll-like receptors
WT: Wild type
