# Supplementary material for: Deletion of Socs3 in LysM+ cells and Cx3cr1 resulted in age-dependent development of retinal microgliopathy
Source: Mol Neurodegener. 2021 Feb 18;16:9. doi: 10.1186/s13024-021-00432-9 (PMC7891019; doi:10.1186/s13024-021-00432-9)
Supplement: Supplementary file 1 — Additional file 1. Genotype of the DKO mice. Genotyping PCRs were performed using primer combinations distinguishing the WT and knockout alleles for LysMCre, Socs3 and Cx3cr1 respectively. (A) Primers used for mouse genotyping. (B) Representative PCR images. For Socs3fl/fl loci, DKO mice showed the band at 700 bp while wild type (WT) sample showed band at 500 bp and the heterozygous showed both bands. For the LysMCre loci, both DKO and heterozygous samples showed band at 700 bp while the WT at 350 bp. DKO mice showed a band of 1200 bp for Cx3cr1 allele, while WT showed band at 970 bp and the heterozygous showed both bands. [file 13024_2021_432_MOESM1_ESM.docx]

Additional file 1. Genotype of the DKO mice. Genotyping PCRs were performed using primer combinations distinguishing the WT and knockout alleles for *LysMCre, Socs3* and *Cx3cr1* respectively. (A) Primers used for mouse genotyping. (B) Representative PCR images. For *Socs3^fl/fl^* loci, DKO mice showed the band at 700 bp while wild type (WT) sample showed band at 500 bp and the heterozygous showed both bands. For the *LysMCre* loci, both DKO and heterozygous samples showed band at 700 bp while the WT at 350 bp. DKO mice showed a band of 1200 bp for *Cx3cr1* allele, while WT showed band at 970 bp and the heterozygous showed both bands.
