# Supplementary material for: Deletion of Socs3 in LysM+ cells and Cx3cr1 resulted in age-dependent development of retinal microgliopathy
Source: Mol Neurodegener. 2021 Feb 18;16:9. doi: 10.1186/s13024-021-00432-9 (PMC7891019; doi:10.1186/s13024-021-00432-9)
Supplement: Supplementary file 2 — Additional file 2. Mouse strain used for each experiment. [file 13024_2021_432_MOESM2_ESM.docx]

Additional file 2. Mouse strain used for each experiment

| Experiment   \| Strain/age \| \| --- \| \| | C57BL/6J | *Socs3^fl/fl^* | | *LysMCre*-*Socs3^fl/fl^* | | *Cx3cr1*^gfp/gfp^ | | DKO | |
| --- | --- | --- | --- | --- | --- | --- | --- | --- | --- | --- |
|  | Young | Young | Aged | Young | Aged | Young | Aged | Young | Aged |
| TEFI |  |  |  |  |  | 🗸 | 🗸 | 🗸 | 🗸 |
| Retinal microglial and neuronal immunofluorescence |  | 🗸 | 🗸 | 🗸 | 🗸 | 🗸 | 🗸 | 🗸 | 🗸 |
| H&E staining |  | 🗸 | 🗸 | 🗸 | 🗸 | 🗸 | 🗸 | 🗸 | 🗸 |
| RPE dismorphology analysis |  |  |  |  |  |  |  |  | 🗸 |
| Phagocytosis, PCR, Luminex* |  | 🗸 |  |  |  |  |  | 🗸 |  |
| Retinal explants* | 🗸 | 🗸 |  |  |  |  |  | 🗸 |  |
