# Supplementary material for: Deletion of Socs3 in LysM+ cells and Cx3cr1 resulted in age-dependent development of retinal microgliopathy
Source: Mol Neurodegener. 2021 Feb 18;16:9. doi: 10.1186/s13024-021-00432-9 (PMC7891019; doi:10.1186/s13024-021-00432-9)
Supplement: Supplementary file 3 — Additional file 3. The death of DKO mice during aging. The life span of DKO mice was recorded during aging. 27 out of 47 mice died by the age of 12 months old. Data showing the percentage of mice died at different ages. [file 13024_2021_432_MOESM3_ESM.docx]

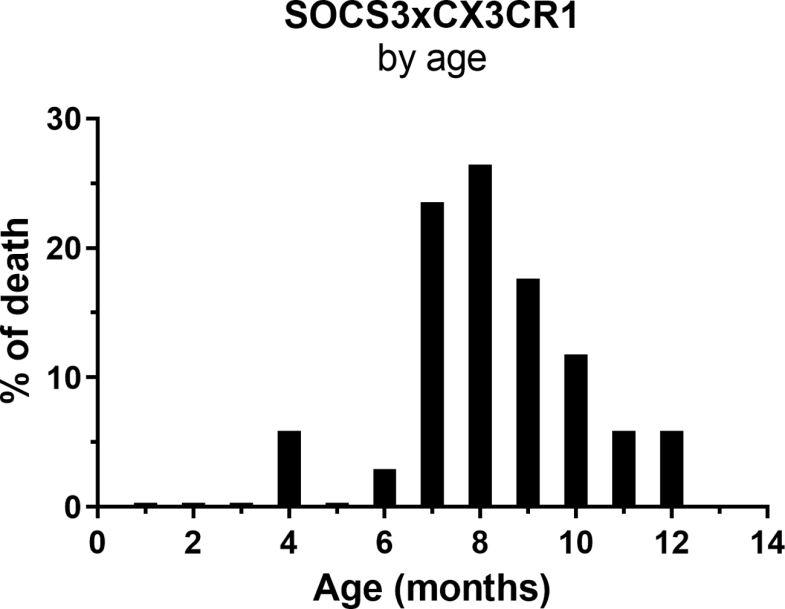


Additional file 3. The death of DKO mice during aging. The life span of DKO mice was recorded during aging. 27 out of 47 mice died by the age of 12 months old. Data showing the percentage of mice died at different ages.
