# Supplementary material for: Deletion of Socs3 in LysM+ cells and Cx3cr1 resulted in age-dependent development of retinal microgliopathy
Source: Mol Neurodegener. 2021 Feb 18;16:9. doi: 10.1186/s13024-021-00432-9 (PMC7891019; doi:10.1186/s13024-021-00432-9)
Supplement: Supplementary file 4 — Additional file 4. Microglial sub-retinal accumulation and Isolectin B4 expression in different strains of young and aged mice. Microglia were labelled by immunostaining of Isolectin B4 (IBA4) in young (3-5m) and aged (10-12m) mice. (A) Representative images showed RPE/choroid flatmounts staining. Microglia are visualised by IBA-1 staining (green) in Socs3fl/fl and LysMCre-Socs3fl/fl mice or the gfp tag in Cx3cr1gfp/gfp and DKO mice. All samples were imaged by Dmi8 fluorescence microscopy (see Materials and Methods). (B) The graph showed quantification of microglia in RPE/choroid flatmounts of different mice. (C) Activated microglia (GFP+) were detected by Isolectin B4 staining in aged DKO retina in both photoreceptor layer and RPE flatmount. Mean ± SD. N ≥ 3 mice, Scale bar: 1000 μm (A) and 50 μm (C). Two-way ANOVA, Tukey’s test, **, P < 0.01. [file 13024_2021_432_MOESM4_ESM.docx]

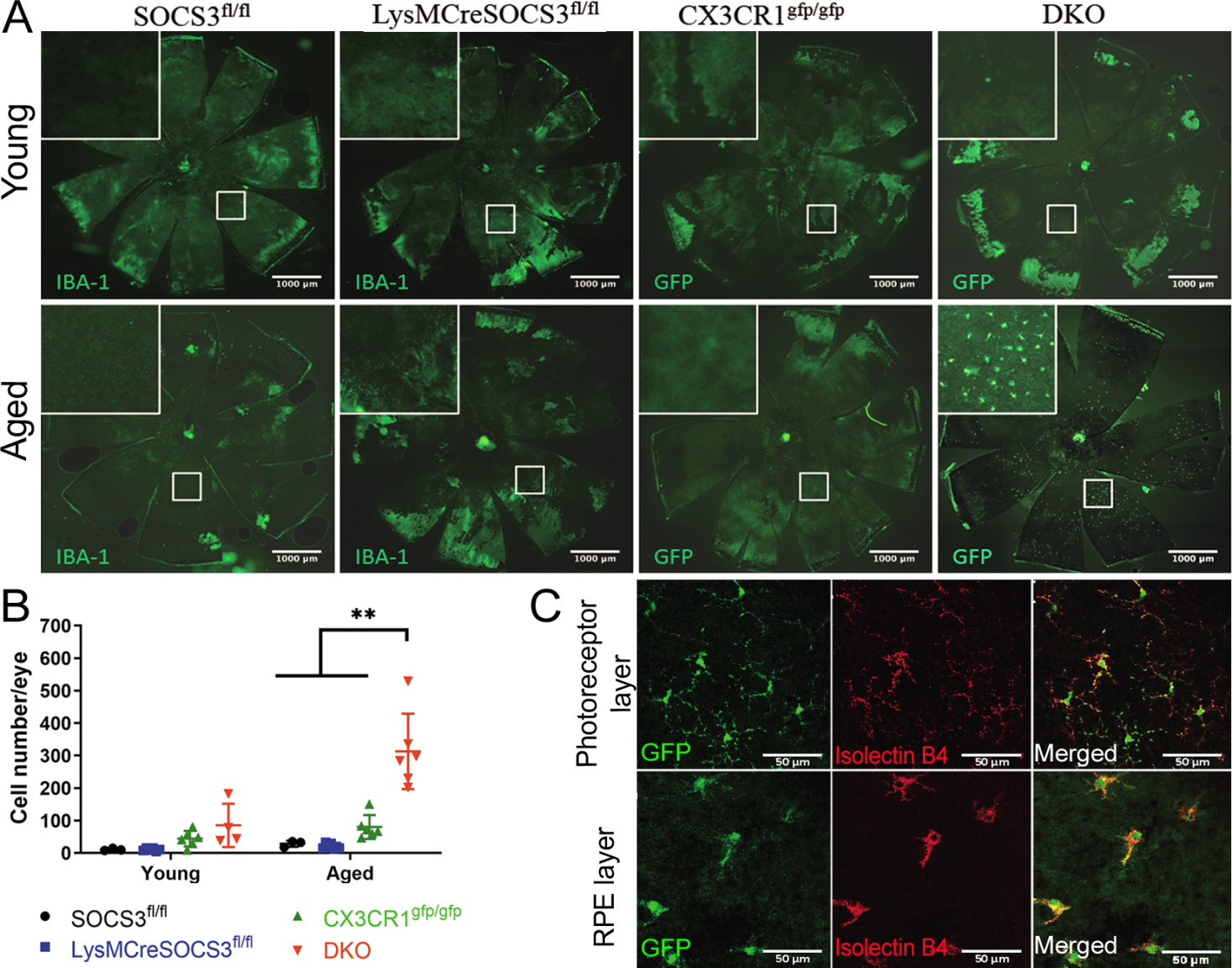


Additional file 4. Microglial sub-retinal accumulation and Isolectin B4 expression in different strains of young and aged mice. Microglia were labelled by immunostaining of Isolectin B4 (IBA4) in young (3-5m) and aged (10-12m) mice. (A) Representative images showed RPE/choroid flatmounts staining. Microglia are visualised by IBA-1 staining (green) in *Socs3^fl/fl^* and *LysMCre-Socs3^fl/fl^* mice or the gfp tag in *Cx3cr1^gfp/gfp^* and DKO mice. All samples were imaged by Dmi8 fluorescence microscopy (see Materials and Methods). (B) The graph showed quantification of microglia in RPE/choroid flatmounts of different mice. (C) Activated microglia (GFP^+^) were detected by Isolectin B4 staining in aged DKO retina in both photoreceptor layer and RPE flatmount. Mean ± SD. N ≥ 3 mice, Scale bar: 1000 µm (A) and 50 µm (C). Two-way ANOVA, Tukey’s test, **, P < 0.01.
