# Supplementary material for: Deletion of Socs3 in LysM+ cells and Cx3cr1 resulted in age-dependent development of retinal microgliopathy
Source: Mol Neurodegener. 2021 Feb 18;16:9. doi: 10.1186/s13024-021-00432-9 (PMC7891019; doi:10.1186/s13024-021-00432-9)
Supplement: Supplementary file 5 — Additional file 5. Electroretinogram (ERG) in LysMCre-Socs3fl/flCx3cr1gfp/gfp DKO mice. Ganzfeld ERG was conducted in young (3 months old) and aged (11 months old) DKO mice with different intensities of flash light. (A) a-wave amplitudes. (B) b-wave amplitudes. (C) Oscillatory potentials. Mean ± SD, n = 8, *P<0.05; **P<0.01; ***P<0.005; ****P<0.001. Two-way ANOVA with Sidak’s multiple comparison test in (A, B). Unpaired Student t test was used in C. [file 13024_2021_432_MOESM5_ESM.docx]

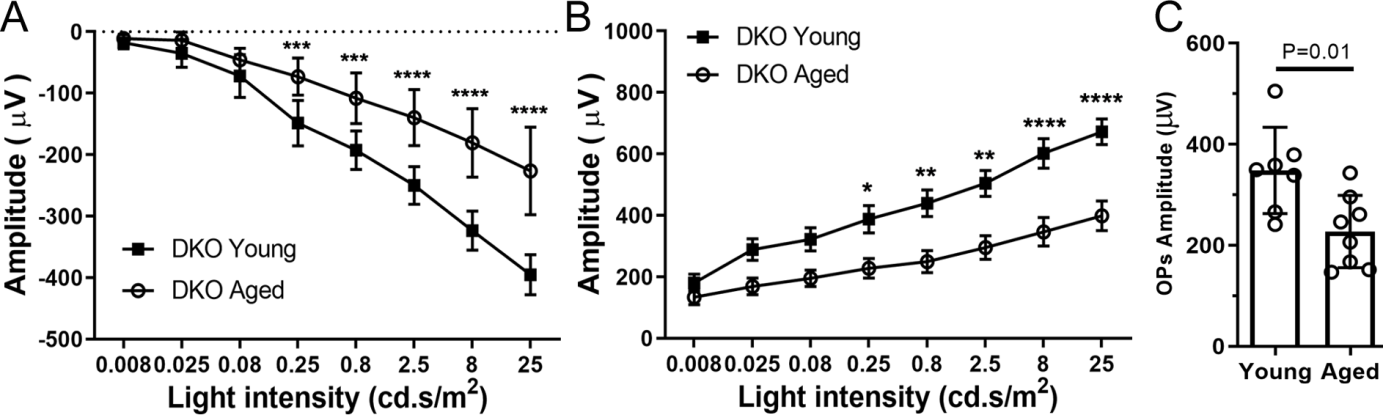


Additional file 5. Electroretinogram (ERG) in *LysMCre-Socs3^fl/fl^Cx3cr1*^gfp/gfp^ DKO mice. Ganzfeld ERG was conducted in young (3 months old) and aged (11 months old) DKO mice with different intensities of flash light. (A) a-wave amplitudes. (B) b-wave amplitudes. (C) Oscillatory potentials. Mean ± SD, n = 8, *P<0.05; **P<0.01; ***P<0.005; ****P<0.001. Two-way ANOVA with Sidak’s multiple comparison test in (A, B). Unpaired Student t test was used in C.
