# Supplementary material for: Deletion of Socs3 in LysM+ cells and Cx3cr1 resulted in age-dependent development of retinal microgliopathy
Source: Mol Neurodegener. 2021 Feb 18;16:9. doi: 10.1186/s13024-021-00432-9 (PMC7891019; doi:10.1186/s13024-021-00432-9)
Supplement: Supplementary file 6 — Additional file 6. GFAP expression in different strains of young and aged mouse retina. Retinal sections from young (3-5m) and aged (10-12m) Socs3fl/fl, LysMCre-Socs3fl/fl, Cx3cr1gfp/gfp and DKO mice were stained with GFAP and DAPI to assess Müller glia activation. (A) Representative images showing GFAP stained of retinal sections from different groups of mice. Scale bar: 50 μm. (B) Quantitative analysis of GFAP+ area in different groups of mice. Two-way ANOVA followed by Sidak’s multiple comparisons. N ≥ 3 mice, *, P < 0.05 compared young and aged DKO; #, P<0.05 between old DKO and old Socs3fl/fl. [file 13024_2021_432_MOESM6_ESM.docx]

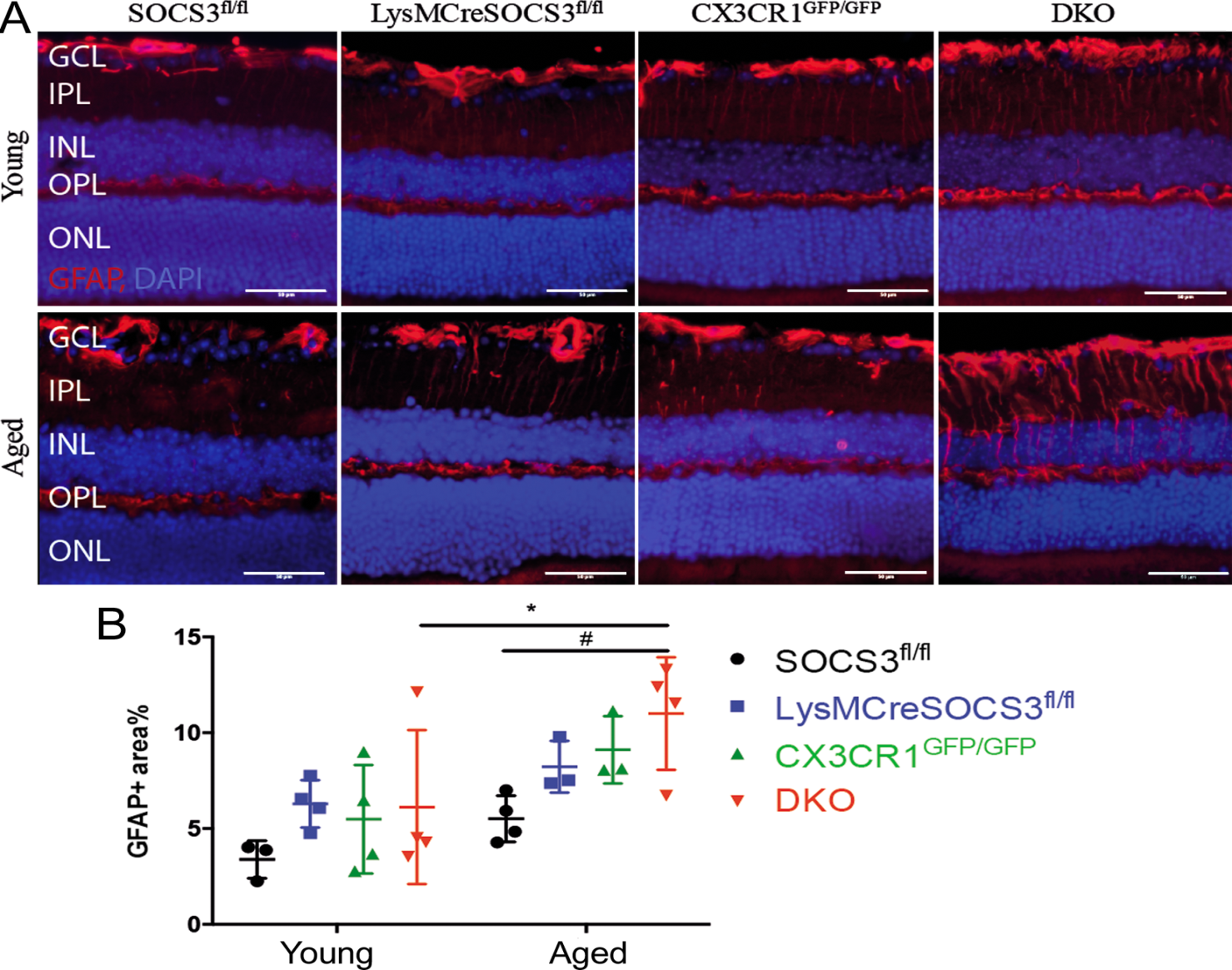


Additional file 6. GFAP expression in different strains of young and aged mouse retina. Retinal sections from young (3-5m) and aged (10-12m) *Socs3^fl/fl^, LysMCre-Socs3^fl/fl^,* *Cx3cr1^gfp/gfp^* and DKO mice were stained with GFAP and DAPI to assess Müller glia activation. (A) Representative images showing GFAP stained of retinal sections from different groups of mice. Scale bar: 50 µm. (B) Quantitative analysis of GFAP^+^ area in different groups of mice. Two-way ANOVA followed by Sidak’s multiple comparisons. N ≥ 3 mice, *, P < 0.05 compared young and aged DKO; ^#^, P<0.05 between old DKO and old SOCS^fl/fl^.
